# Polybromo 1/vimentin axis dictates tumor grade, epithelial-mesenchymal transition, and metastasis in pancreatic cancer

**DOI:** 10.1172/JCI177533

**Published:** 2025-06-02

**Authors:** Munenori Kawai, Akihisa Fukuda, Munehiro Ikeda, Kei Iimori, Kenta Mizukoshi, Kosuke Iwane, Go Yamakawa, Mayuki Omatsu, Mio Namikawa, Makoto Sono, Tomonori Masuda, Yuichi Fukunaga, Munemasa Nagao, Osamu Araki, Takaaki Yoshikawa, Satoshi Ogawa, Yukiko Hiramatsu, Motoyuki Tsuda, Takahisa Maruno, Yuki Nakanishi, Dieter Saur, Tatsuaki Tsuruyama, Toshihiko Masui, Etsuro Hatano, Hiroshi Seno

**Affiliations:** 1 Department of Gastroenterology and Hepatology and; 2 Department of Drug Discovery Medicine, Medical Innovation Center, Kyoto University Graduate School of Medicine, Kyoto, Japan.; 3 Department of Internal Medicine II, Klinikum Rechts der Isar, Technische Universität München, Munich, Germany.; 4 Division of Translational Cancer Research, German Cancer Research Center (DKFZ) and German Cancer Consortium (DKTK), Heidelberg, Germany.; 5 Division of Hepato-Biliary-Pancreatic Surgery and Transplantation, Department of Surgery, Kyoto University Graduate School of Medicine, Kyoto, Japan.

**Keywords:** Gastroenterology, Oncology, Cancer, Epigenetics, Mouse models

## Abstract

Mutations in Polybromo 1 (*PBRM1*), a subunit of the switch/sucrose nonfermentable (SWI/SNF) chromatin remodeling complex, are frequently observed in several cancers, including pancreatic ductal adenocarcinoma (PDAC). In this study, we demonstrated that pancreas-specific loss of *Pbrm1* in mice harboring *Kras* mutations and *Trp53* deletions accelerated the development of poorly differentiated PDAC, epithelial-mesenchymal transition (EMT), and metastasis, resulting in worsened prognosis. *Pbrm1* loss in preexisting PDAC shifted the tumor grade from a well- to a poorly differentiated state and elevated vimentin expression. *Pbrm1*-null PDAC exhibited downregulation of apical junction genes and upregulation of EMT pathway genes, including the vimentin and squamous molecular subtype signature genes. Mechanistically, PBRM1 bound to the vimentin gene promoter and directly downregulated its expression. Furthermore, suppression of vimentin in *Pbrm1*-null PDAC cells reversed the dedifferentiation phenotype and reduced EMT and metastasis. Consistently, reduced PBRM1 expression correlated with high vimentin expression, poorly differentiated histology, a high recurrence rate, and reduced overall survival in human PDACs. Additionally, PDAC with *PBRM1* deletion was associated with the aggressive squamous molecular subtype. Our data established PBRM1 as a tumor suppressor that controls tumor grade and metastasis of PDAC by regulating vimentin expression.

## Abstract 2

Polybromo 1 (PBRM1) is a tumor suppressor that controls tumor grade and metastasis of pancreatic ductal adenocarcinoma by regulating vimentin expression.

## Graphical abstract

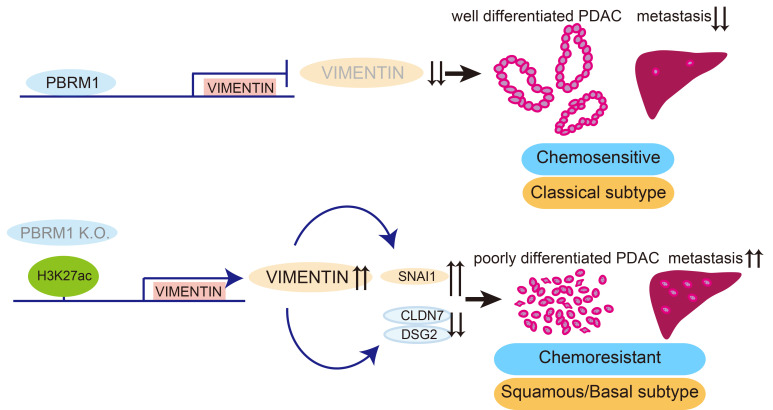

## Introduction

Pancreatic ductal adenocarcinoma (PDAC) is a lethal disease with a 5-year survival rate of 12% (1). At the time of diagnosis, a mere 10%–15% of patients present with localized (2, 3) diseases that may be resectable with curative intent. Approximately 30%–35% of the patients have locally advanced tumors, while the remaining 50%–55% of the patients display metastatic lesions (4). At present, it is considered that surgical resection is needed to eradicate PDAC; however, approximately 80% of PDAC cases recur within 5 years after resection, while over 60% of PDAC patients experience recurrence within 2 years (5). This clinical course suggests that most patients may already harbor undetectable micrometastases at the time of resection. Furthermore, the leading cause of death among cancer patients is the direct or indirect effects of metastatic disease (6). Therefore, effective antimetastatic treatment strategies are needed. Consequently, a comprehensive understanding of the molecular mechanisms underlying PDAC development, metastasis, and local advancement of PDAC is crucial.

The tumor grade is a measure of the degree of tumor differentiation. It measures the resemblance of malignant cells to the morphological and functional characteristics of the tissue of origin (7). A high tumor grade in PDAC is considered a risk factor for early recurrence after resection (8–10), a predictor of reduced postrecurrence survival (8), and an indicator of overall survival after resection (11–13). However, the molecular mechanism underlying tumor grade has not been fully elucidated.

Recently, transcriptomic subtyping of PDAC was performed. Collisson et al. documented 3 subtypes (classical, quasimesenchymal, and exocrine-like) (14), Bailey et al. documented four subtypes (immunogenic, progenitor, ADEX, and squamous) (15), while Moffitt et al. reported 2 subtypes (classical and basal-like) (16). Although the subtyping details differed between these studies, they largely overlapped. The squamous, quasimesenchymal, and basal-like subtypes were similar, and all 3 were associated with a poor prognosis in these studies.

Recent genome-wide sequencing studies have shown that mutations in the subunit genes of switch/sucrose nonfermentable (SWI/SNF) chromatin-remodeling complexes are widespread in various human cancers (17, 18) and have been observed in 12%–23% of human PDACs (17, 19, 20). SWI/SNF complexes convert chromatin architectures using energy from ATP hydrolysis to regulate gene expression (21).

The SWI/SNF comprises 29 subunits encoded by each gene, which assemble into 3 distinct SWI/SNF complexes, including canonical BRG1/BRM–associated factor (BAF), polybromo-associated BAF (PBAF), and non-canonical BAF (ncBAF). Polybromo 1 (PBRM1) is a PBAF-specific subunit, and inactivating mutations in *PBRM1* have been reported in several cancers, including PDAC. These mutations account for approximately 5% of PDAC cases (20). PBRM1 functions in the specific recruitment of SWI/SNF complex by binding to various genes (22). However, the role of PBRM1 in the initiation and progression of PDAC remains unclear.

In this study, we evaluated the impact of PBRM1 loss in the pancreas using a mouse PDAC model harboring *Kras* mutation and *Trp53* deletion through conditional deletion of *Pbrm1*. We observed that pancreas-specific PBRM1 loss significantly accelerated the formation of poorly differentiated and undifferentiated carcinoma and increased distant metastasis with a worse prognosis. Furthermore, loss of PBRM1 in preexisting PDAC caused a shift in the tumor grade from a well- to a poorly differentiated state, concomitant with increased vimentin expression. Notably, suppression of vimentin expression in *Pbrm1*-null PDAC cells reversed the dedifferentiation phenotype and reduced metastasis. We demonstrated that the PBRM1/vimentin axis is a critical regulator of tumor grade and metastasis in both mouse and human PDAC.

## Results

### Low PBRM1 expression is associated with high tumor grade, high recurrence rate, poor prognosis, and the squamous molecular subtype of human PDAC.

**Figure 1:**
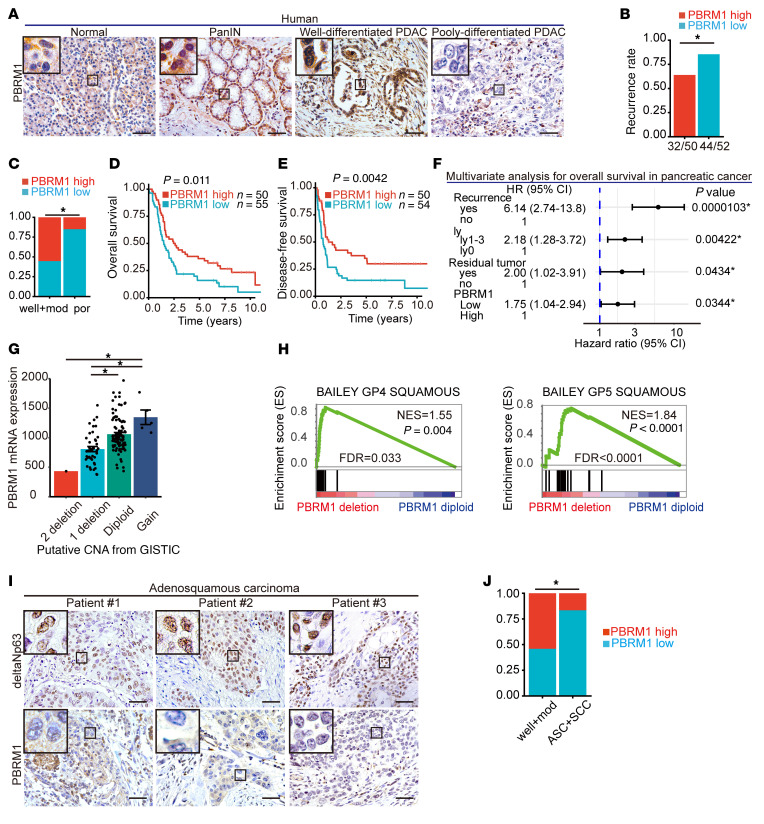
Low PBRM1 expression is associated with high tumor grade, high recurrence rate, poor prognosis, and the squamous molecular subtype. (**A**) Representative immunohistochemical analysis (IHC) of PBRM1 in human pancreatic samples. Scale bar: 50 μm. Data are representative of 3 independent experiments. (**B**) Recurrence rates at high (*n* = 50) and low (*n* = 52) PBRM1 expression levels in human PDACs. (**C**) Rates of high and low PBRM1 IHC levels in human well- and moderately differentiated pancreatic ductal adenocarcinoma (well+mod) (*n* = 85) and poorly differentiated pancreatic ductal adenocarcinoma (por) (*n* = 20). (**D** and **E**) Kaplan-Meier plots showing overall survival or disease-free survival in a cohort of pancreatic cancer patients with high (*n* = 50) and low (*n* = 55 (**D**), 54 (**E**)) PBRM1 protein expression levels. (**F**) Multivariate Cox proportional hazard analysis for overall survival in a cohort of patients with pancreatic cancer. HR, hazard ratio. CI, confidence interval. (**G**) *PBRM1* mRNA expression with each putative copy number alteration status from a cohort of 147 patients in TCGA dataset. (**H**) GSEA enrichment plots of BAILEY GP4 SQUAMOUS and BAILEY GP5 SQUAMOUS in PDAC tumors from *PBRM1* diploid PDAC (*n* = 100) and PDAC with *PBRM1* deletion (*n* = 42) in a cohort of 142 patients in TCGA dataset. NES, normalized enrichment score; FDR, false discovery rate. (**I**) Representative IHC results of ΔNp63 and PBRM1 in human adenosquamous carcinoma samples (*n* = 11). Scale bar: 50 μm. (**J**) Rates of high and low PBRM1 IHC levels in human well- and moderately differentiated PDAC (well+mod) (*n* = 85) and adenosquamous carcinoma (ASC) (*n* = 11) and squamous cell carcinoma of the pancreas (SCC) (*n* = 1). *P < 0.05. **G**, Data shown as mean ± SE. **B**, Pearson’s χ^2^ test. **C** and **J**, Fisher’s exact test. **D** and **E**, Log-rank (Mantel-Cox) test. **G**, 1-way ANOVA, followed by the Tukey’s multiple comparison test.

To determine the expression pattern of PBRM1 in human pancreatic epithelial cells, we performed IHC analysis. PBRM1 was expressed in ductal cells and the majority of acinar cells in the normal pancreas (Figure 1A). Further IHC analysis in 105 surgically resected specimens of PDAC revealed high PBRM1 expression in 50 cases and low PBRM1 expression in 55 cases (Supplemental Figure 1A; supplemental material available online with this article; https://doi.org/10.1172/JCI177533DS1). Subsequently, correlations between the expression levels of PBRM1 and clinicopathological features were examined. Tumor recurrence and grade were significantly correlated with PBRM1 expression (Table 1). Low PBRM1 expression was associated with a high recurrence rate (85%), poorly differentiated histology, and short overall and disease-free survival (Figure 1, A–E). Univariate Cox proportional hazards analysis revealed that several parameters, including tumor grade, expression level of PBRM1, tumor recurrence, residual tumor, N category, and lymphatic invasion were significant predictors of overall survival in patients with PDAC (Supplemental Table 1). Among these parameters, recurrence, expression level of PBRM1, N category, and lymphatic invasion were determined to be independent predictors by multivariate Cox proportional hazards analysis (Figure 1F and Supplemental Table 2).

An inquiry into the Cancer Genome Atlas-Pancreatic Adenocarcinoma (TCGA-PAAD) cohort (20) for mutations and Genomic Identification of Significant Targets in Cancer (GISTIC) putative copy number alterations (CNAs) (23) revealed frequent heterozygous loss of *PBRM1*. Out of 147 cases, one patient exhibited a homozygous deletion of *PBRM1*, while 41 of them exhibited a heterozygous deletion of *PBRM1*. The CNA data revealed that the group with *PBRM1* deletion demonstrated significantly lower expression of *PBRM1* mRNA in comparison with the group without *PBRM1* CNA (Figure 1G). Gene set enrichment analysis (GSEA) was conducted using molecular subtyping gene sets (14–16) for PDAC with *PBRM1* deletion and *PBRM1* diploid PDAC. It was observed that squamous gene sets were significantly enriched in the PDAC with *PBRM1* deletion (Figure 1H). The basal-like gene set, the quasimesenchymal gene set, and PID ΔNp63 pathway gene set were also enriched in the PDAC with *PBRM1* deletion, although it did not reach statistical significance (Supplemental Figure 1B). In the TCGA-PAAD cohort, we found that the proportion of patients classified to basal-like, squamous, or quasimesenchymal subtype was larger in the PDAC with *PBRM1* deletion than that in the PDAC with *PBRM1* diploid, although it did not reach statistical significance (Supplemental Figure 1C).

Analysis of the TCGA data revealed that the expression of genes upregulated in the squamous molecular subtype of human PDAC, including *CKS1B, HSPE1, EIF2S2, KIF18A, UBE2C, CKAP2L,* and *CCNA2*, was also upregulated in human PDAC with *PBRM1* deletion in comparison with *PBRM1*-diploid PDAC (Supplemental Figure 1D). Additionally, the deltaNp63 protein, which is a typical squamous/basal marker, was strongly expressed, and the PBRM1 protein expression was significantly lower in resected pancreatic squamous cell carcinoma and adenosquamous carcinoma specimens than in well- or moderately differentiated PDAC specimens (Figure 1, I and J). Ten out of 12 samples of human pancreatic adenosquamous carcinoma or squamous cell carcinoma lost the PBRM1 expression. All the human PDAC samples except one that we analyzed (*n* = 24) showed negative expression of ΔNp63 isoform (Supplemental Figure 1E). We also evaluated 6 resected specimens of undifferentiated carcinoma of the pancreas by immunostaining of PBRM1. Five of the 6 pancreatic undifferentiated carcinoma specimens had low PBRM1 expression, a rate higher than that of well- or moderately differentiated PDAC (Supplemental Figure 1, F and G). Collectively, these data demonstrate that PBRM1 expression is inversely associated with high tumor grade, high recurrence rate, poor prognosis, and the squamous molecular subtype in human PDAC.

### Pancreatic Pbrm1 deletion accelerates the formation of precancerous lesions and leads to poorly differentiated PDAC with a poor prognosis.

Subsequently, we determined the localization of PBRM1 expression in mouse pancreatic epithelial cells. Similar to human samples, PBRM1 expression was observed in ductal cells and the majority of acinar cells in the pancreas of WT mice (Supplemental Figure 2A). PBRM1 was also ubiquitously expressed in the pancreatic intraepithelial neoplasia (PanIN) and PDAC of *Ptf1a^Cre^; LSL-Kras^G12D^* (*KC*) and *Ptf1a^Cre^; LSL-Kras^G12D^;* and *Trp53^f/wt^* (*KPC*) mice (Supplemental Figure 2A).

To investigate the functional requirements of PBRM1 during pancreatic development, we crossed transgenic mice carrying floxed alleles of *Pbrm1* (*Pbrm1^f^*) with *Ptf1a^Cre^* (24) (*C*) mice. *Ptf1a^Cre^; Pbrm1^f/f^* (*CPb^–/–^*) *Ptf1a^Cre^;* and *Pbrm1^f/wt^* (*CPb^+/–^*) mice were born as predicted by the Mendelian ratio and were indistinguishable from control *Ptf1a^Cre^* mice in terms of pancreatic appearance, pancreas/body weight ratio, and histology at 6 weeks of age (Supplemental Figure 2, B and C). PBRM1 loss in *CPb^–/–^* acinar cells was confirmed by IHC analysis (Supplemental Figure 2C) and qRT-PCR of acinar cells isolated from *CPb^–/–^* and control *C* pancreata (Supplemental Figure 2D). Notably, IHC analysis revealed a decreased number of PBRM1-positive acinar cells in *CPb^+/–^* pancreata compared with control *C* pancreata. Additionally, qRT-PCR revealed reduced expression of *Pbrm1* in acinar cells isolated from *CPb^+/–^* pancreata in comparison with control *C* pancreata (Supplemental Figure 2D). These data indicated that pancreatic PBRM1 is dispensable for pancreatic development in mice.

**Figure 2:**
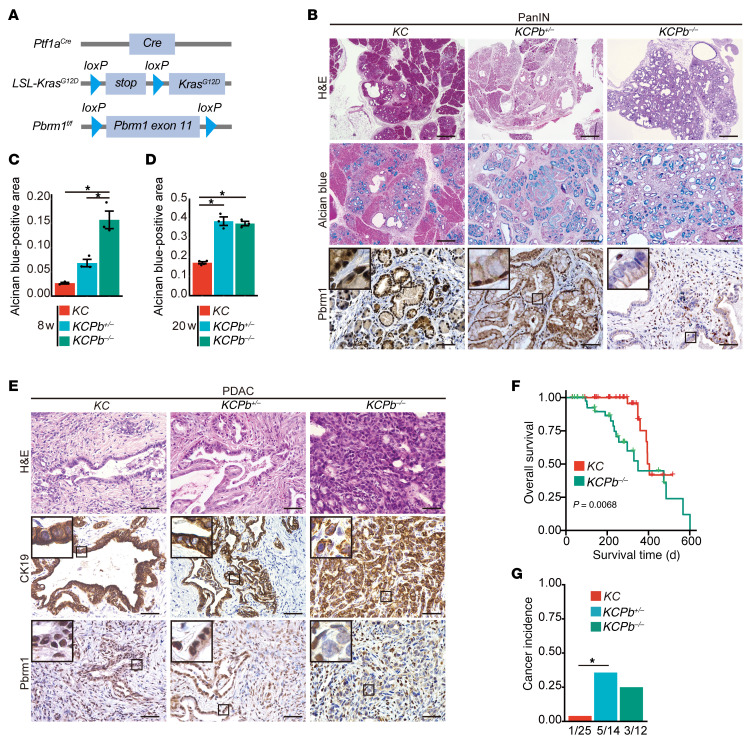
Pancreatic PBRM1 deletion accelerates the formation of precancerous lesions and leads to poorly differentiated pancreatic ductal adenocarcinoma with a poor prognosis. (**A**) The genetic strategy employed to activate oncogenic *Kras* and delete *Pbrm1,* specifically in the pancreas, obtained from the embryonic stage. (**B**) Representative hematoxylin and eosin (H&E), Alcian blue, and PBRM1 staining in *Ptf1a^Cre^; LSL-Kras^G12D^*(*KC*), *Ptf1a^Cre^; LSL-Kras^G12D^; Pbrm1^f/wt^* (*KCPb^+/–^*), and *Ptf1a^Cre^; LSL-Kras^G12D^; Pbrm1^f/f^* (*KCPb^–/–^*) mice at 20 weeks of age. Scale bar: 200 μm (H&E and alcian blue); 50 μm (Pbrm1). Data are representative of 3 independent experiments. (**C**) Quantification of Alcian blue–positive late acinar-to-ductal metaplasia and PanINs determination by combining 3 independent sections from *KC* (*n* = 3), *KCPb^+/–^* (*n* = 3), and *KCPb^–/–^* (*n* = 3) mice at 8 weeks of age. *P < 0.05, 1-way ANOVA, followed by Tukey’s multiple comparison test. Data are shown as mean ± SE. (**D**) Quantification of Alcian blue–positive late acinar-to-ductal metaplasia and PanINs determination by combining 3 independent sections from *KC* (*n* = 4), *KCPb^+/–^* (*n* = 3), and *KCPb^–/–^* (*n* = 3) mice at 20 weeks of age. *P < 0.05, 1-way ANOVA, followed by Tukey’s multiple comparison test. Data are shown as mean ± SE. (**E**) Representative H&E, CK19, and PBRM1 staining in PDACs from *KC*, *KCPb^+/–^*, and *KCPb^–/–^* mice. Scale bar: 50 μm. Data are representative of 3 independent experiments. (**F**) Kaplan-Meier plots showing the overall survival in the cohorts of *KC* (*n* = 86) and *KCPb^–/–^* (*n* = 54) mice. The log-rank (Mantel-Cox) test was used to assess statistical significance. (**G**) Rate of PDAC incidence in *KC* (*n* = 25), *KCPb^+/–^* (*n* = 14)*,* and *KCPb^–/–^* (*n* = 12) mice aged 20–30 weeks. *P < 0.05, Fisher’s exact test.

Next, we investigated the effect of pancreatic *Pbrm1* loss on pancreatic tumorigenesis driven by oncogenic *Kras*. We generated *Ptf1a^Cre^, LSL-Kras^G12D^,* and *Pbrm1^f/f^* (*KCPb^–/–^*) mice and compared them with control *Ptf1a^Cre^* and *LSL-Kras^G12D^* (*KC*) mice (Figure 2A). Histologically, at 4, 8, 12, and 20 weeks of age, duct-like structures were observed in *KCPb^–/–^* pancreata that were more prominent than in *KC* pancreata (Figure 2B and Supplemental Figure 3A). This finding suggested that *Pbrm1* loss resulted in exacerbations of metaplastic ductal changes in the context of oncogenic *Kras*. Consistent with this finding, at 8 and 20 weeks of age, *KCPb^–/–^* pancreata demonstrated a significant increase in Alcian blue–positive PanIN areas in comparison with *KC* pancreata (Figure 2, C and D). Notably, *KCPb^+/–^* pancreata demonstrated a significant increase in Alcian blue–positive PanIN areas in comparison with *KC* pancreata at 20 weeks of age. We scored for regions of ADM and PanIN1/2/3 in *KC*, *KCPb^+/–^*, and *KCPb^–/–^* mice pancreata at 20 weeks of age and found *KCPb^+/–^* and *KCPb^–/–^* mice had increased areas of higher grades of PanIN and decreased areas of the normal acinar compared with *KC* mice pancreata (Supplemental Figure 3B). IHC analysis confirmed that PBRM1 was deleted in *KCPb^–/–^* pancreata (Figure 2B). These data indicate that the pancreas-specific heterozygous or homozygous deletion of *Pbrm1* markedly accelerates the formation of PanIN in the context of oncogenic *Kras*. During a long-term observation period spanning between 20 and 30 weeks of age, a small number of *KCPb^–/–^* mice developed PDAC. Histologically, the PDACs observed in *KCPb^–/–^* pancreata were poorly differentiated adenocarcinomas (Figure 2E). Furthermore, we observed significantly reduced survival in *KCPb^–/–^* mice in comparison to control *KC* mice (Figure 2F). We also evaluated the survival of *KCPb^+/–^* mice. The survival of *KCPb^+/–^* mice was intermediate in those of *KC* and *KCPb^–/–^* mice (Supplemental Figure 3C). We analyzed mice without invasive carcinoma at the moribund state and found that the mice appeared to die of pancreatic exocrine insufficiency and malabsorption. Pancreatic exocrine insufficiency and malabsorption were observed in *KCPb^–/–^* mice but not in *KC* mice, as determined by positive oil red O staining of stool and the decreased pancreas/body weight ratio (Supplemental Figure 3, D and E). We performed coimmunostaining of CK19 and vimentin in PDAC from *KC*, *KCPb^+/–^,* and *KCPb^–/–^* mice at the moribund state. We found that PDACs in *KCPb^–/–^* mice at the moribund state had more mesenchymal features than those in *KC* and *KCPb^+/–^* mice in terms of a higher rate of vimentin-positive cancer cells (Supplemental Figure 3, F–H). PDACs in *KCPb^+/–^* mice at the moribund had somewhat mesenchymal features, but they were less mesenchymal than those in *KCPb^–/–^* mice (Supplemental Figure 3, F–H).

*Ptf1a^Cre^; LSL-Kras^G12D^; Pbrm1^f/wt^* (*KCPb^+/–^*) mice demonstrated a moderate phenotype between *KC* and *KCPb^–/–^* mice concerning PanIN formation, tumor grade, and survival (Figure 2, C–E). Notably, *KCPb^+/–^* mice exhibited a significantly higher incidence of PDAC than *KC* mice (Figure 2G). On the other hand, *KCPb^–/–^* mice demonstrated a higher but statistically insignificant incidence of PDAC than *KC* mice.

These results indicate that PBRM1 plays a haploinsufficient tumor-suppressive role in PDAC formation in the context of oncogenic *Kras*.

### Pancreatic Pbrm1 loss synergizes with oncogenic Kras and heterozygous Trp53 deletion to yield poorly differentiated PDAC and induce liver metastasis with a poor prognosis.

**Figure 3:**
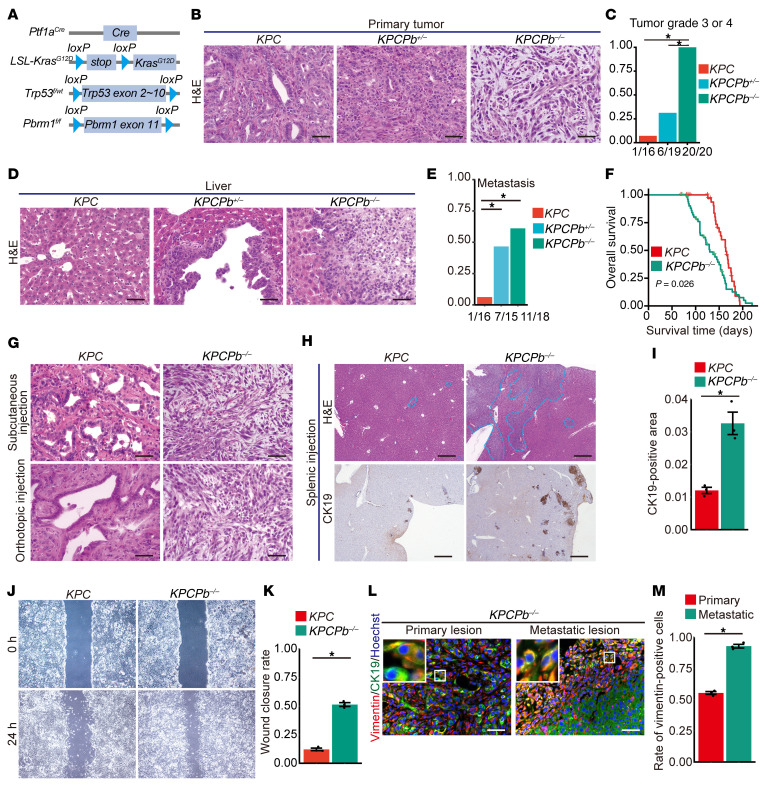
Pancreatic PBRM1 loss synergizes with oncogenic KRAS and heterozygous *Trp53* deletion to yield poorly differentiated PDAC and induce liver metastasis with a poor prognosis. (**A**) Genetic strategy used to activate oncogenic *Kras* and delete *Pbrm1* and *Trp53* specifically in the pancreas from the embryonic stage. (**B**) Representative H&E staining in PDAC from *Ptf1a^Cre^; LSL-Kras^G12D^; Trp53^f/wt^* (*KPC*) (*n* = 16), *Ptf1a^Cre^; LSL-Kras^G12D^; Trp53^f/wt^; Pbrm1^f/wt^* (*KPCPb^+/–^*) (*n* = 18) and *Ptf1a^Cre^; LSL-Kras^G12D^; Trp53^f/wt^; Pbrm1^f/f^* (*KPCPb^–/–^*) (*n* = 20) mice at the primary site. Scale bar: 50 μm. (**C**) Rate of tumor grade 3 or 4 of PDACs in *KPC* (*n* = 16), *KPCPb^+/–^* (*n* = 19), and *KPCPb^–/–^* (*n* = 20) mice. (**D**) Representative H&E staining in the livers of *KPC* (*n* = 16) mice and metastatic PDAC in the livers of *KPCPb^+/–^* (*n* = 7) and *KPCPb^–/–^* (*n* = 11) mice. Scale bar: 50 μm. (**E**) Quantification of liver metastasis incidence in *KPC* (*n* = 16), *KPCPb^+/–^* (*n* = 15)*,* and *KPCPb^–/–^* (*n* = 18) mice during moribund state. (**F**) Kaplan-Meier plots showing overall survival of *KPC* (*n* = 37) and *KPCPb^–/–^* (*n* = 52) mice. (**G**) Representative H&E staining in PDAC allografted subcutaneously or orthotopically with PDAC cells from *KPC* (*n* = 3) and *KPCPb^–/–^* (*n* = 3) mice. Scale bar: 50 μm. (**H**) Representative H&E and CK19 staining of liver metastases after injection of PDAC cells into the spleen of *KPC* (*n* = 3) and *KPCPb^–/–^* (*n* = 3) mice. Metastatic lesions were circled by blue lines in H&E staining. Scale bar: 500 μm. (**I**) Rate of CK19-positive areas determined by combining 3 independent sections of liver metastases after injection of PDAC cells into the spleen of *KPC* (*n* = 3) and *KPCPb^–/–^* (*n* = 3) mice. (**J**) Representative image of the scratch assay with PDAC cells from *KPC* (*n* = 3) and *KPCPb^–/–^* (*n* = 3) mice. (**K**) Quantification of the scratch assay using PDAC cells from *KPC* (*n* = 3) and *KPCPb^–/–^* (*n* = 3) mice. (**L**) Representative coimmunostaining of vimentin, CK19, and Hoechst in primary and metastatic lesions in *KPCPb^–/–^* mice (*n* = 3). Scale bar: 50 μm. (**M**) Quantification of the rate of the vimentin-positive cancer cells in primary and metastatic lesions in *KPCPb^–/–^* mice (*n* = 3). *P < 0.05. **C,** **E,** Fisher’s exact test. **F,** Log-rank (Mantel-Cox) test. **I, K** Student *t* test. **M,** paired *t* test. Data shown as mean ± SE.

To further investigate the effects of *Pbrm1* loss on PDAC formation in the context of heterozygous *Trp53* deletion, we generated *Ptf1a^Cre^; LSL-Kras^G12D^; Trp53^f/wt^; Pbrm1^f/f^* (*KPCPb^–/–^*), and *Ptf1a^Cre^; LSL-Kras^G12D^; Trp53^f/wt^; Pbrm1^f/wt^* (*KPCPb^+/–^*) mice, and compared them with the control *Ptf1a^Cre^; LSL-Kras^G12D^; Trp53^f/wt^* (*KPC*) mice (Figure 3A). *KPCPb^–/–^* mice developed poorly differentiated PDAC more frequently than control *KPC* mice and *KPCPb^+/–^* (Figure 3, B and C and Supplemental Figure 4A). Additionally, *KPCPb^–/–^* mice developed undifferentiated pancreatic carcinoma. H&E staining of PDAC that developed in *KPCPb^–/–^* mice displayed spindle and multinuclear cells with scant cytoplasm, indistinct cell borders, and hyperchromatic nuclei, which are characteristics of undifferentiated pancreatic carcinoma (Figure 3B and Supplemental Figure 4B). We scored for regions of well-, moderately, and poorly differentiated PDAC, and undifferentiated carcinoma in *KPC*, *KPCPb^+/–^*, and *KPCPb^–/–^* mice pancreata at the moribund state and found *KPCPb^+/–^* and *KPCPb^–/–^* mice pancreata had increased areas of poorly-differentiated adenocarcinoma and decreased areas of well-differentiated adenocarcinoma compared with *KPC* mouse pancreata (Supplemental Figure 4C). Notably, *KPCPb^–/–^* and *KPCPb^+/–^* mice exhibited liver metastases more frequently than *KPC* mice, as determined by H&E staining and CK19 immunostaining of mice liver (Figure 3, D and E and Supplemental Figure 4D). We observed significantly reduced survival in *KPCPb^–/–^* mice than in control *KPC* mice (Figure 3F). Immunostaining revealed that some PDACs in *KPCPb^–/–^* mice were positively stained for p63, a typical marker of the squamous subtype (15), whereas PDACs in *KPC* mice did not show staining for p63 (Supplemental Figure 4E). Subcutaneous or orthotopic tumors allografted with mouse PDAC cells obtained from *KPCPb^–/–^*, *KPCPb^+/–^*, and *KPC* mice revealed tumor grades comparable with the original cells (Figure 3G). Splenic injection of PDAC cells from *KPCPb^–/–^* mice resulted in a significantly elevated metastatic tumor burden in comparison with control PDAC cells from *KPC* mice (Figure 3H). Immunostaining revealed a substantial increase in cytokeratin19–positive (CK19-positive) metastatic areas in the livers of mice transplanted with *KPCPb^–/–^* PDAC cells in comparison with those of control mice (Figure 3I). Consistent with the in vivo data, the scratch assay revealed that cancer cells from *KPCPb^–/–^* mice exhibited higher migratory activity than those from *KPC* mice (Figure 3, J and K). Furthermore, PDAC cells from *KPCPb^–/–^* mice exhibited mesenchymal-like morphology in cell culture dishes (Supplemental Figure 4E).

We performed coimmunofluorescence of metastatic lesions of the *KPCPb^–/–^* mouse model and splenic injection mouse model with the antibody of vimentin and CK19. As a result, most of the metastatic lesions were more mesenchymal than each primary lesion in terms of a higher rate of vimentin-positive cancer cells (Figure 3, L and M). These data suggest that *Pbrm1-*null metastatic PDAC cells underwent a positive selection of EMT during pancreatic cancer progression.

These results indicate that pancreatic *Pbrm1* loss synergizes with oncogenic *Kras* and heterozygous *Trp53* deletion to yield poorly differentiated PDAC or undifferentiated carcinoma of the pancreas with squamous subtype properties, and induces liver metastasis, resulting in a poor prognosis. Therefore, *Pbrm1*-deficient PDAC exhibits higher aggressiveness in terms of tumor grade, metastatic potential, and prognosis in the context of heterozygous *Trp53* deletion.

### Pancreatic Pbrm1 loss synergizes with oncogenic Kras and homozygous Trp53 deletion to accelerate the development of poorly differentiated PDAC and to facilitate the EMT of PDAC cells resulting in a poor prognosis.

**Figure 4:**
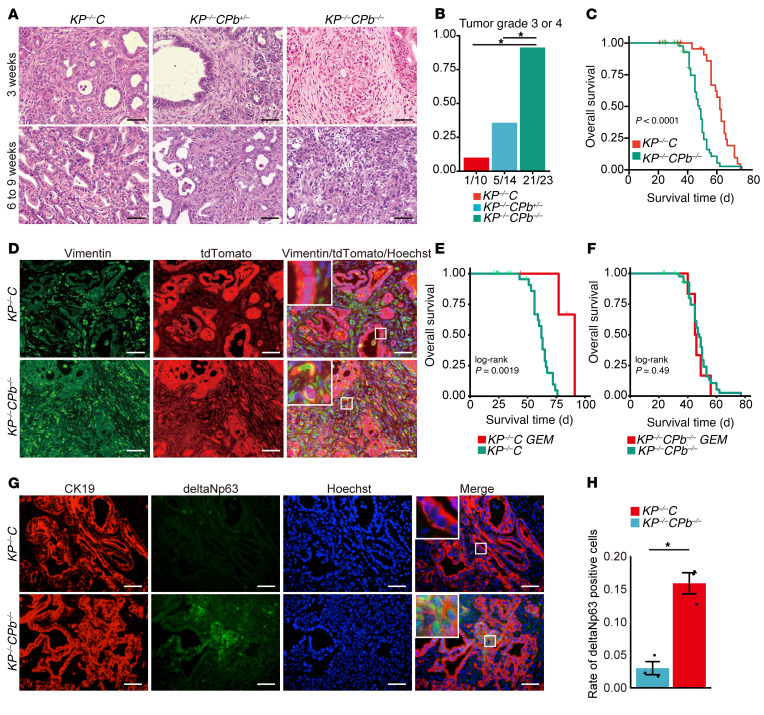
Pancreatic PBRM1 loss synergizes with oncogenic KRAS and homozygous *Trp53* deletion to accelerate the development of poorly differentiated PDAC and to facilitate the EMT of PDAC cells, resulting in a poor prognosis. (**A**) Representative H&E staining of the pancreas from *Ptf1a^Cre^; LSL-Kras^G12D^; Trp53^f/f^* (*KP^–/–^C*), *Ptf1a^Cre^; LSL-Kras^G12D^; Trp53^f/f^; Pbrm1^f/wt^* (*KP^–/–^CPb^+/–^*), and *Ptf1a^Cre^; LSL-Kras^G12D^; Trp53^f/f^; Pbrm1^f/f^* (*KP^–/–^CPb^–/–^*) mice at 3 weeks of age and 6-to-9 weeks of age in the moribund state. Scale bar: 50 μm. Data are representative of 3 independent experiments. (**B**) Rate of tumor grade 3 or 4 of PDACs in *KP^–/–^C* (*n* = 10), *KP^–/–^CPb^+/–^* (*n* = 14), and *KP^–/–^CPb^–/–^* (*n* = 23) mice. *P < 0.05, Fisher’s exact test. (**C**) Kaplan-Meier plots showing overall survival of *KP^–/–^C* (*n* = 31) and *KP^–/–^CPb^–/–^* (*n* = 51) mice. The log-rank (Mantel-Cox) test has been used to assess the statistical significance. (**D**) Representative coimmunostaining of vimentin and tdTomato in PDAC from *Ptf1a^Cre^; LSL-Kras^G12D^ Trp53^f/f^*; *LSL-Rosa^td–tomato^* (*KP^–/–^CTomato*) (*n* = 3) and *Ptf1a^Cre^; LSL-Kras^G12D^; Trp53^f/f^; Pbrm1^f/f^*; *LSL-Rosa^td–tomato^* (*KP^–/–^CPb^–/–^Tomato*) (*n* = 3) mice. Scale bar: 50 μm. (**E**) Kaplan-Meier plots showing overall survival of *KP^–/–^C* mice treated with gemcitabine (*n* = 4) and *KP^–/–^C* mice without treatment with gemcitabine (*n* = 31). The log-rank (Mantel-Cox) test has been used to assess the statistical significance. (**F**) Kaplan-Meier plots showing overall survival of *KP^–/–^CPb^–/–^* mice treated with gemcitabine (*n* = 6) and *KP^–/–^CPb^–/–^* mice without treatment with gemcitabine (*n* = 51). The log-rank (Mantel-Cox) test has been used to assess the statistical significance. (**G**) Representative coimmunostaining of CK19 and ΔNp63 in PDAC from *KP^–/–^C* (*n* = 3) and *KP^–/–^CPb^–/–^* (*n* = 3) mice. Scale bar: 50 μm. (**H**) Quantification of the rate of the ΔNp63-positive cancer cells in PDAC from *KP^–/–^C* (*n* = 3) and *KP^–/–^CPb^–/–^* (*n* = 3) mice. *P < 0.05, Student *t* test. Data shown as mean ± SE.

To further investigate the effects of *Pbrm1* loss on PDAC formation in the context of homozygous *Trp53* deletion, we generated *Ptf1a^Cre^; LSL-Kras^G12D^; Trp53^f/f^; Pbrm1^f/f^* (*KP^–/–^CPb^–/–^*), and *Ptf1a^Cre^; LSL-Kras^G12D^; Trp53^f/f^; Pbrm1^f/wt^* (*KP^–/–^CPb^+/–^*) mice and compared them with control *Ptf1a^Cre^; LSL-Kras^G12D^ Trp53^f/f^* (*KP^–/–^C*) mice (Supplemental Figure 5A). The *KP^–/–^C* model with homozygous *Trp53* deletion displays a rapidly progressing primary tumor with a shorter latency of tumorigenesis and poorer survival than the *KPC* model with heterozygous *Trp53* deletion (25). Histologically, *KP^–/–^CPb^–/–^* mice developed poorly differentiated PDAC significantly more frequently compared with *KP^–/–^C* mice (Figure 4, A and B and Supplemental Figure 5B). Furthermore, *KP^–/–^CPb^–/–^* mice developed poorly differentiated PDAC with aggressive infiltrative growth as early as 3 weeks of age (Figure 4A). Moreover, pancreatic cells in these mice were completely replaced by invasive PDAC lesions in all *KP^–/–^CPb^–/–^* mice (*n* = 6) when analyzed at 5 weeks of age. *KP^–/–^CPb^–/–^* mice exhibited a higher pancreatic tumor burden than control *KP^–/–^C* mice at both 3 and 5 weeks of age (Supplemental Figure 5C). Immunostaining revealed a significant reduction in amylase-positive areas in the *KPCPb^–/–^* pancreata compared with those in the control *KP^–/–^C* pancreata (Supplemental Figure 5, D and E). At 5 weeks of age, approximately one-quarter of the pancreatic cells were histologically normal in *KP^–/–^C* mice (Supplemental Figure 5, D and E). Even in the moribund stage at over 8 weeks of age, *KP^–/–^C* mice exhibited some histologically normal pancreatic cells (Supplemental Figure 5F), indicating that oncogenic *Kras* and homozygous *Trp53* deletion were not enough to transform all pancreatic acinar cells into cancerous or precancerous cells, but oncogenic Kras and homozygous Trp53 deletion plus homozygous *Pbrm1* deletion were. These findings suggest that the loss of PBRM1 accelerates KRAS-driven tumorigenesis, even in the *Trp53-*null background. We observed significantly reduced survival in *KP^–/–^CPb^–/–^* mice compared with control *KP^–/–^C* mice (Figure 4C). Analysis of metastasis was not feasible in this model because most of the mice died from local invasion or advancement, resulting in jaundice.

These results indicate that pancreatic *Pbrm1* loss also synergizes with oncogenic *Kras* and homozygous *Trp53* deletion to form poorly differentiated PDAC with short latency and rapid growth, resulting in a poor prognosis.

*Pbrm1*-null PDACs exhibit a mesenchymal appearance with poor differentiation and are characterized by high metastatic potential. Therefore, to determine the impact of PBRM1 loss on EMT during PDAC development in vivo, we performed lineage tracing experiments. To track the fate of the mutated pancreatic epithelial cells, we generated *Ptf1a^Cre^; LSL-Kras^G12D^; Trp53^f/f^; Pbrm1^f/f^*, *LSL-Rosa^td–tomato^* (*KP^–/–^CPb^–/–^Tomato*) mice and compared them with the control *Ptf1a^Cre^; LSL-Kras^G12D^ Trp53^f/f^*; *LSL-Rosa^td–tomato^* (*KP^–/–^CTomato*) mice (Supplemental Figure 5G). In these animals, Cre recombination induced tdTomato expression in all cells originating from *Ptf1a*-expressing pancreatic progenitor cells during the fetal period (*i.e*., pancreatic epithelial cells). In control *KP^–/–^CTomato* mice, the majority of tdTomato-positive (red) cells were negative for the mesenchymal markers (vimentin and fibronectin). In contrast, in *Pbrm1-*null PDACs, tdTomato-positive (red) cells were positive for the mesenchymal markers (vimentin and fibronectin) (Figure 4D and Supplemental Figure 5H). These data indicate that the mesenchymal cells in *Pbrm1*-null PDACs originate from pancreatic epithelial cells through EMT and that pancreatic PBRM1 loss promotes EMT.

### Pbrm1-deficient PDACs are resistant to chemotherapy and associated with the squamous molecular subtype.

To examine chemosensitivity of *Pbrm1*-null PDAC, we performed treatment with gemcitabine in the *Pbrm1*-WT and *Pbrm1*-null PDAC mouse models. As a result, *KP^–/–^C* mice showed markedly longer survival when treated with gemcitabine, whereas *KP^–/–^CPb^–/–^* mice had no survival benefit from treatment with gemcitabine. These data indicate that *Pbrm1*-deficient PDAC represented an aggressive feature of resistance to chemotherapy similar to that of squamous subtype pancreatic cancer in humans (Figure 4, E and F).

To see whether mouse *Pbrm1*-null PDACs associated with the aggressive squamous molecular subtype in human PDACs, we next performed coimmunofluorescence of *Pbrm1*-WT and *Pbrm1*-null PDACs with the antibody of ΔNp63 isoform and CK19. We found that *Pbrm1* loss correlated with the expression of the ΔNp63 isoform in mice (Figure 4, G and H). These data indicate that mouse *Pbrm1*-null PDACs associated with the aggressive squamous molecular subtype, which was consistent with human data.

### Pancreatic acinar cell–specific ablation of PBRM1 in the context of oncogenic KRAS activation and heterozygous Trp53 deletion induces poorly differentiated PDAC.

In our previous studies, we reported that the role of BRG1, an ATPase subunit of the SWI/SNF complex, in PDAC initiation, is cell-type dependent. BRG1 has a tumor-suppressive role in pancreatic ductal cells, but a tumor-promoting role in pancreatic acinar cells (26, 27). Therefore, to further determine the effects of PBRM1 loss specifically in pancreatic acinar cells, we subsequently generated *Ptf1a^CreER^; LSL-Kras^G12D^; Trp53^f/wt^; Pbrm1^f/f^* (*ER-KPCPb^–/–^*) mice and compared them with control *Ptf1a^CreER^; LSL-Kras^G12D^ Trp53^f/wt^* (*ER-KPC*) and *Ptf1a^CreER^; LSL-Kras^G12D^; Trp53^f/wt^; Pbrm1^f/wt^* (*ER-KPCPb^+/–^*) mice, permitting expression of mutant *Kras*, *Pbrm1* deletion and heterozygous *Trp53* deletion, specifically, in adult acinar cells upon tamoxifen treatment (Supplemental Figure 6A). PBRM1 loss was confirmed in *ER-KPCPb^–/–^* pancreata through IHC analysis (Supplemental Figure 6B). Histologically, *ER-KPCPb^–/–^* mice developed poorly differentiated PDAC and undifferentiated carcinoma, similar to *KPCPb^–/–^* mice, and some *ER-KPCPb^–/–^* mice exhibited liver metastases (Supplemental Figure 6B). PDACs that developed in *ER-KPCPb^–/–^* mice exhibited higher vimentin expression than those in *ER-KPC* mice (Supplemental Figure 6B).

These results indicate that PBRM1 plays a tumor-suppressive role in mature acinar cells, as observed in *Ptf1a*-expressing pancreatic progenitor cells.

### PBRM1 ablation in established PDAC results in the conversion of tumor grade into poorly differentiated PDAC in mice.

**Figure 5:**
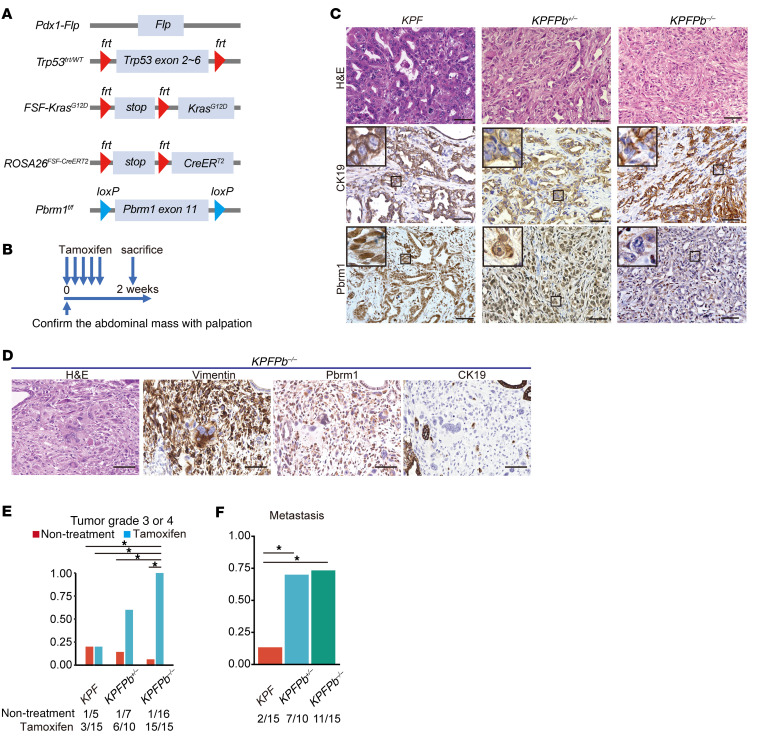
PBRM1 ablation in established PDAC results in the conversion of tumor grade into poorly differentiated PDAC in mice. (**A**) Genetic strategy used to activate oncogenic *Kras* and delete *Trp53* heterozygously at the embryonic stage and delete *Pbrm1* at the tumor-bearing adult stage. (**B**) Experimental design for tamoxifen administration and analysis. (**C**) Representative H&E, CK19, and PBRM1 staining in PDAC from *Pdx1-Flp; FSF-Kras^G12D^; Trp53^fr/wt^; FSF-Rosa26^CAG–CreERT2^* (*KPF*), *Pdx1-Flp; FSF-Kras^G12D^; Trp53^fr/wt^; FSF-Rosa26^CAG–CreERT2^; Pbrm1^f/wt^* (*KPFPb^+/–^*) and *Pdx1-Flp; FSF-Kras^G12D^; Trp53^fr/wt^; FSF-Rosa26^CAG–CreERT2^; Pbrm1^f/f^* (*KPFPb^–/–^*) mice 2 weeks after tamoxifen administration. Scale bar: 50 μm. Data are representative of 3 independent experiments. (**D**) Representative H&E, vimentin, PBRM1, and CK19 staining of PDAC from *KPFPb^–/–^* mice 2 weeks after tamoxifen administration, which exhibited a transient state of degradation of the tubular component to undifferentiated carcinoma. Scale bar: 50 μm. Data are representative of 3 independent experiments. (**E**) Rate of tumor grade 3 or 4 of PDACs in *KPF* (*n* = 15)*, KPFPb^+/–^* (*n* = 10)*,* and *KPFPb^–/–^* (*n* = 15) mice 2 weeks after tamoxifen administration and PDACs in *KPF* (*n* = 5)*, KPFPb^+/–^* (*n* = 7)*,* and *KPFPb^–/–^* (*n* = 16) mice without tamoxifen administration. *P < 0.05, Fisher’s exact test. (**F**) Rate of liver metastasis in *KPF* (*n* = 15), *KPFPb^+/–^* (*n* = 10)*,* and *KPFPb^–/–^* (*n* = 15) mice 2 weeks after tamoxifen administration. *P < 0.05, Fisher’s exact test.

In the PDAC mouse models described above, *Pbrm1* loss occurred simultaneously with the activation of oncogenic *Kras* and the deletion of *Trp53*, which prompted us to investigate whether *Pbrm1* deletion in established PDAC has any precise effects on PDAC tumor cells. To address this question, we used a dual-recombinase system, which permits independent temporal modification of both alleles of target genes using flippase (Flp) and Cre recombinases (28). In this mouse model, we activated oncogenic *Kras* and heterozygously deleted *Trp53* using Flp recombinase directed by the mouse *Pdx1* promoter. To delete *Pbrm1* in Flp-recombinant cells via Cre recombinase at a later time point, we used a tamoxifen-inducible *CreERT2* allele silenced by an *frt-stop-frt (FSF)* cassette at the *Rosa26* locus. Tamoxifen treatment induced expression of the *CreERT2* allele, which, in turn, induced recombination of the *loxP* site at the *Pbrm1* locus (Figure 5A). *Pbrm1* was deleted by tamoxifen administration after ensuring PDAC formation by palpation in mice at 12–16 weeks of age in *Pdx1-Flp; FSF-Kras^G12D^; Trp53^frt/wt^; FSF-ROSA26^CreERT2^; Pbrm1^f/f^* *(KPFPb^–/–^)* mice. Two weeks after tamoxifen administration, the animals were euthanized and their pancreata were analyzed (Figure 5B). At the 2-week time point, control (*KPF*) animals developed well-differentiated PDACs, whereas *Pbrm1*-null mice exhibited significantly more poorly differentiated PDACs or undifferentiated carcinomas of the pancreas than control mice (Figure 5, C–E). Loss of *Pbrm1* expression in PDACs developed in *Pbrm1*-null mice was confirmed by IHC analysis (Figure 5C). Some *Pbrm1*-deleted PDAC cells exhibited a transient state of degradation of the tubular component to a component of undifferentiated carcinoma with high vimentin expression and loss of expression of PBRM1 and CK19 (Figure 5D). Pancreatic heterozygous or homozygous *Pbrm1*-deleted mice demonstrated significantly increased metastasis compared with control mice (Figure 5F).

These results indicated that PBRM1 ablation in established PDAC converted the tumor grade from well-differentiated to poorly differentiated PDAC or undifferentiated carcinoma of the pancreas in mice. Therefore, PBRM1 is a critical determinant of tumor grade not only before PDAC development but also after PDAC formation, and PBRM1 plays a tumor-suppressive role in PDAC progression.

### Pbrm1 loss downregulates the expression of apical junction genes and upregulates the expression of EMT-related genes in PDAC cells.

**Figure 6:**
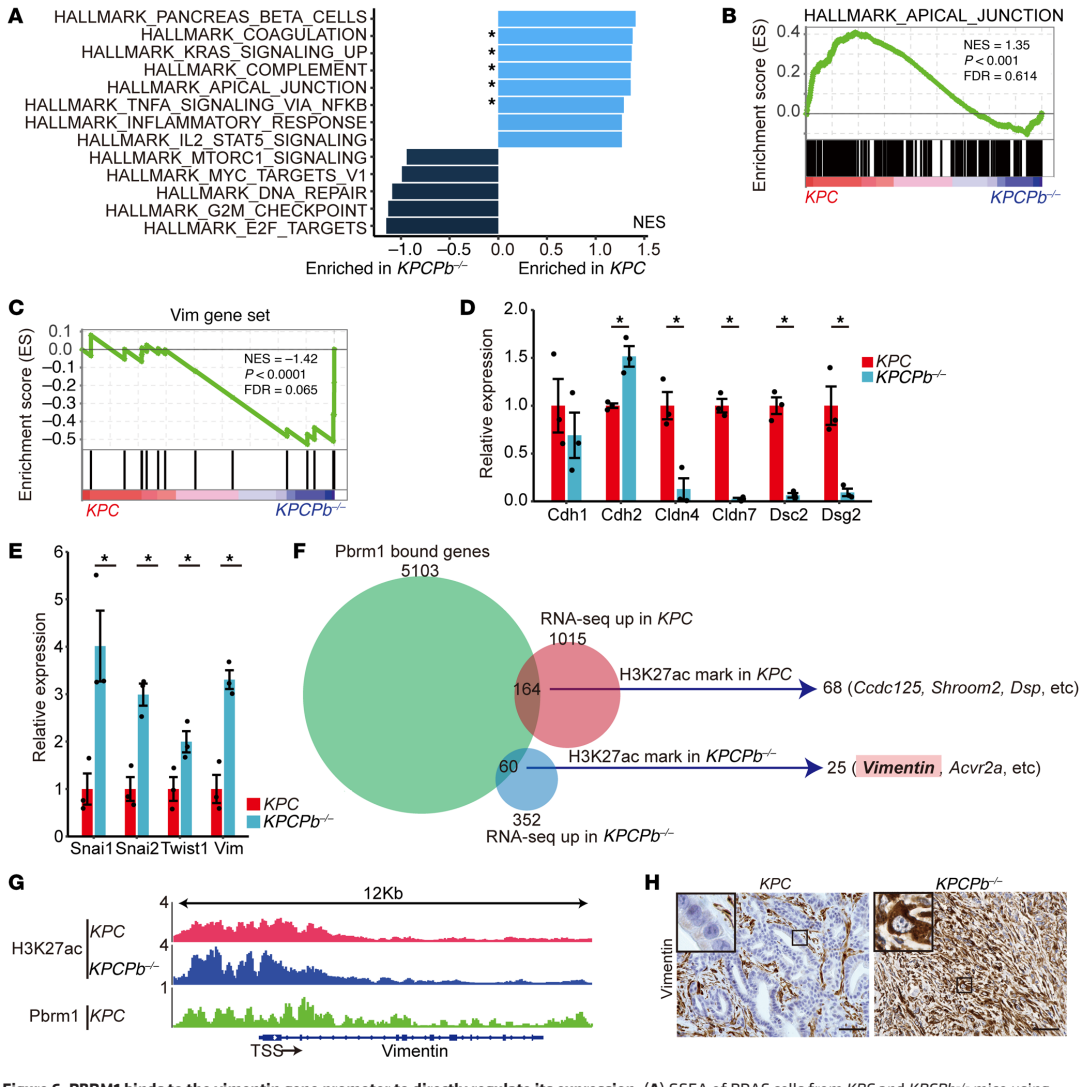
PBRM1 binds to the vimentin gene promoter to directly regulate its expression. (**A**) GSEA of PDAC cells from *KPC* and *KPCPb^–/–^* mice using “Hallmark gene sets.” NES, normalized enrichment score. *P < 0.05. (**B**) GSEA enrichment plots of the HALLMARK apical junction. FDR, false discovery rate. (**C**) GSEA of PDAC cells from *KPC* and *KPCPb^–/–^* mice using the vimentin gene set. (**D**) Quantitative real-time PCR analysis of the relative mRNA expression of *Cdh1, Cdh2, Cldn4, Cldn7, Dsc2,* and *Dsg2* in *KPCPb^–/–^* (*n* = 3) PDAC cells compared with *KPC* (*n* = 3) PDAC cells. *P < 0.05, Student *t* test. Data shown as mean ± SE. (**E**) Quantitative real-time PCR analysis of the relative mRNA expression of *Vim*, *Snai1, Snai2*, and *Twist1* in *KPCPb^–/–^* (*n* = 3) PDAC cells compared with *KPC* (*n* = 3) PDAC cells. *P < 0.05, Student *t* test. Data shown as mean ± SE. (**F**) Venn diagram of the analysis of genes bound by PBRM1 and differentially expressed genes identified by RNA-seq. Sixty-eight genes are bound by H3K27Ac out of the 164 genes that are bound by PBRM1 and upregulated in *KPC* PDAC cells and 25 genes are bound by H3K27ac out of 60 genes that are bound by PBRM1 and upregulated in *KPCPb^–/–^* PDAC cells. (**G**) ChIP data of the PBRM1 and H3K27ac binding region in the vimentin gene promoter and coding regions. TSS, transcription start site. (**H**) Representative vimentin staining in PDAC of *KPC* and *KPCPb^–/–^* mice. Scale bar: 50 μm. Data are representative of 3 independent experiments.

For a comprehensive understanding of the downstream transcriptional effects of *Pbrm1* deletion in the pancreas, we performed RNA-seq on PDAC cells established from *KPCPb^–/–^* (*n* = 3) and *KPC* (*n* = 3) mice. Notably, 1,367 genes were differentially expressed, of which 352 were upregulated and 1,015 were downregulated. Gene set enrichment analysis (GSEA) using “hallmark gene sets” showed that apical junction genes were enriched in PDAC cells from *KPC* mice compared with PDAC cells from *KPCPb^–/–^* mice (Figure 6, A and B). GSEA revealed statistically significant enrichment of the vimentin gene set (29), containing 17 genes cooccurring with the biological term “vim” in literature-supported statements describing the functions of genes from the GeneRIF Biological Term Annotations dataset. This enrichment was observed in PDAC cells from *KPCPb^–/–^* mice when compared with PDAC cells from *KPC* mice (Figure 6C).

Next, we focused on the expression of genes important for the EMT or apical junctions. Quantitative real-time PCR analysis revealed that the expression of tight junction genes, including *Cldn4* and *Cldn7*, and desmosome genes, including *Dsc2* and *Dsg2,* was remarkably downregulated, while the expression of EMT-related genes, including *Vim*, *Snai1, Snai2*, and *Twist1,* which regulate apical junction genes (30), was upregulated in *Pbrm1*-null PDAC cells (Figure 6, D and E). These results indicate that *Pbrm1*-null PDAC exhibited decreased expression of apical junction genes and increased expression of EMT pathway genes. Given that *Pbrm1*-null PDAC cells coexpressed both epithelial (*Cdh1*) and mesenchymal (*Vim*) markers, it was considered that they were in a partial-EMT state (Figure 6, D and E).

Given that human PDAC with *PBRM1* deletion is associated with the squamous molecular subtype of PDAC, we examined the expression of squamous molecular subtype signature genes. In corroboration with the human data, the expression of genes upregulated in the squamous molecular subtype of human PDAC, including *CKS1B, HSPE1, EIF2S2, KIF18A, UBE2C, CKAP2L,* and *CCNA2* was upregulated in PDAC cells from *KPCPb^–/–^* mice, although the difference was not statistically significant (data not shown).

These data are consistent with the finding that *PBRM1*-null PDAC exhibited more aggressive features in terms of tumor grade, metastasis, and squamous molecular subtype.

### PBRM1 binds to the vimentin gene promoter to directly regulate its expression.

Given that PBRM1 is a component of the SWI/SNF chromatin remodeling complex, we next sought to determine the effects of *Pbrm1* loss on the chromatin status of mouse PDAC cells. Using the same *Pbrm1*-null or *Pbrm1*-WT PDAC cells used for RNA-seq analysis, ChIP-seq was performed against PBRM1 and histone 3 lysine 27 acetylation (H3K27ac), which is a well-known marker of active enhancers and promoters. ChIP-seq analysis using an antibody against PBRM1 identified 5,103 genes, of which chromatin occupancies were present in the cis-regulatory regions identified using GREAT (31) in *KPC* PDAC cells (Figure 6F). ChIP-seq analysis using an antibody against H3K27ac identified significantly decreased chromatin occupancy at 1,654 genes and increased chromatin occupancy at 3,126 genes in *KPCPb^–/–^* PDAC cells in comparison with control *KPC* PDAC cells.

We thoroughly examined the genes with PBRM1 peaks and compared them with differentially expressed genes using RNA-seq. We found 164 genes with PBRM1 peaks whose mRNA expression was upregulated in *KPC* PDAC cells, and 60 genes with PBRM1 peaks whose mRNA expression was upregulated in *KPCPb^–/–^* PDAC cells (Figure 6F). Subsequently, 164 and 60 genes were analyzed using H3K27ac marks. Sixty-eight out of 164 genes were upregulated in their expression with H3K27ac peaks in *KPC* cells, including *Dsp*, *Ccdc125*, and *Shroom2* (Figure 6F and Supplemental Table 3). Twenty-five out of 60 genes, including the vimentin gene, were upregulated in their expression with H3K27ac peaks in *KPCPb^–/–^* PDAC cells (Figure 6, F and G and Supplemental Table 3). Furthermore, vimentin protein expression was significantly elevated in the PDAC of *KPCPb^–/–^* mice in comparison with that in control mice, as determined by IHC analysis (Figure 6H). Our results align with previous studies where analysis of ChIP data from GSM4932331 (32) revealed that PBRM1 bound to the cis-regulatory elements of the vimentin gene in the human rhabdomyosarcoma cell line, RH-4 (Supplemental Figure 6C).

These results indicate that PBRM1 binds to the promoter of the vimentin gene to directly downregulate its expression and that *Pbrm1* loss results in elevated expression of vimentin concomitant with upregulation of H3K27ac histone marker at the vimentin promoter in PDAC cells.

### Vimentin inhibition reverses the dedifferentiation phenotype and decreases metastasis of Pbrm1-null PDAC in mice.

**Figure 7:**
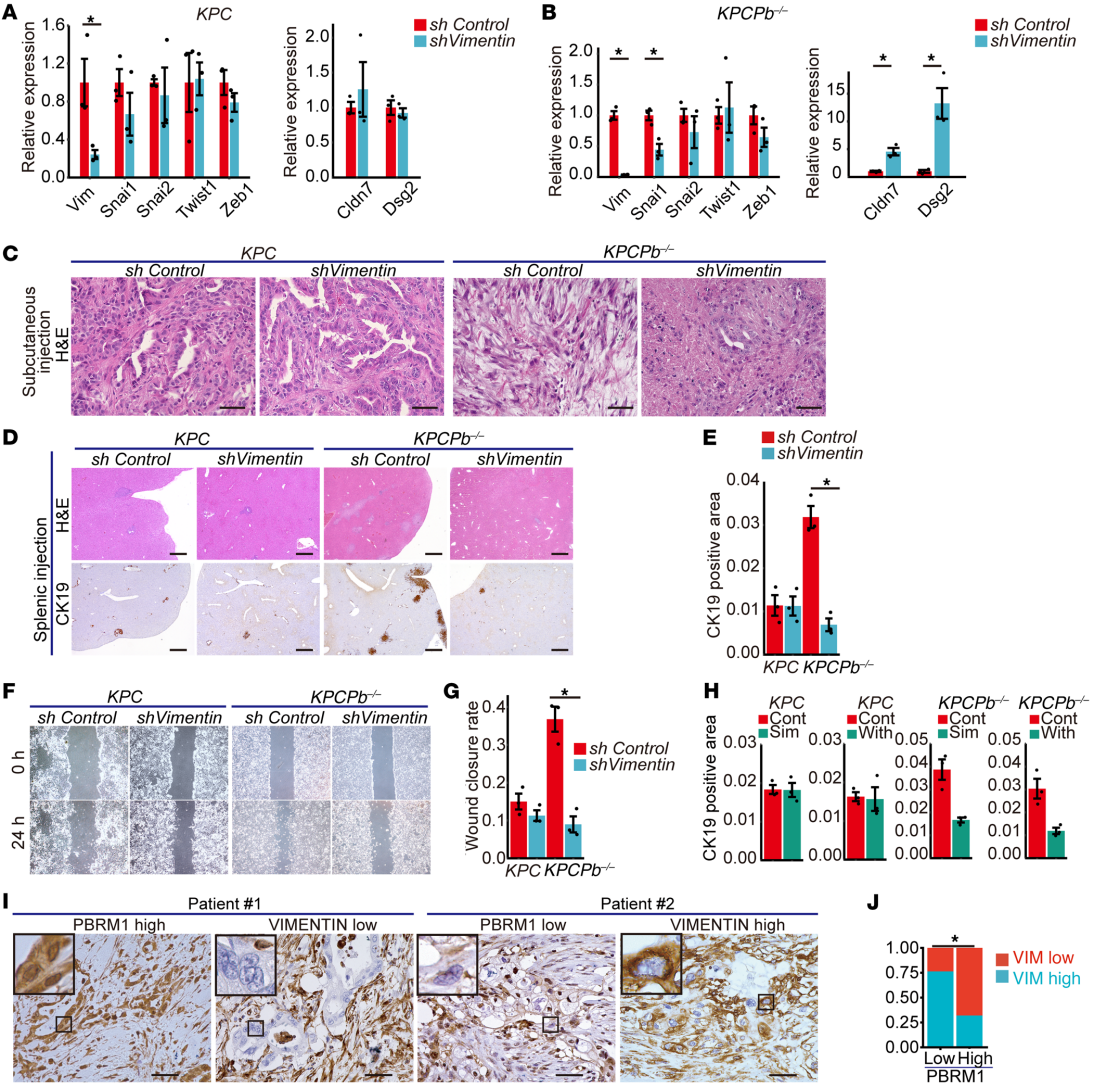
Vimentin inhibition reverses the dedifferentiation phenotype and reduces metastasis of *Pbrm1*-null PDAC in mice. (**A**) Quantitative real-time PCR analysis of the relative mRNA expression of *Zeb1*, *Vim*, *Snai1, Snai2, Twist1, Cldn7,* and *Dsg2* in *KPC* PDAC cells with shRNA knockdown of the vimentin gene (*shVimentin*) (*n* = 3) compared with *KPC* PDAC cells with shRNA control (*shControl*) (*n* = 3). *P < 0.05, Student *t* test. Data shown as mean ± SE. (**B**) Quantitative real-time PCR analysis of the relative mRNA expression of *Zeb1*, *Vim*, *Snai1, Snai2, Twist1, Cldn7,* and *Dsg2* in *KPCPb^–/–^* PDAC cells with shRNA knockdown of the vimentin gene (*shVimentin*) (*n* = 3) compared with *KPCPb^–/–^* PDAC cells with shRNA control (*shControl*) (*n* = 3). *P < 0.05, paired *t* test. Data shown as mean ± SE. (**C**) Representative H&E staining in PDACs allografted subcutaneously with *KPCshControl*, *KPCshVimentin, KPCPb^–/–^shControl*, and *KPCPb^–/–^shVimentin* PDAC cells. Scale bar: 50 μm. Data are representative of 3 independent experiments. (**D**) Representative H&E and CK19 staining in metastatic PDAC after injection into the spleen with *KPCshControl*, *KPCshVimentin, KPCPb^–/–^shControl*, and *KPCPb^–/–^shVimentin* PDAC cells. Scale bar: 500 μm. Data are representative of 3 independent experiments. (**E**) Quantification of CK19-positive liver metastasis with splenic injection of *KPC shControl* (*n* = 3), *KPCshVimentin* (*n* = 3)*, KPCPb^–/–^shControl* (*n* = 3), and *KPCPb^–/–^shVimentin* (*n* = 3) PDAC cells, determined by combining 3 independent sections. *P < 0.05, Student *t* test. Data are represented as mean ± SE. (**F**) Representative images of the scratch assay with *KPC shControl*, *KPCshVimentin, KPCPb^–/–^shControl*, and *KPCPb^–/–^shVimentin* PDAC cells. Data are representative of 3 independent experiments. (**G**) Quantification of the scratch assay with *KPC shControl* (*n* = 3), *KPCshVimentin* (*n* = 3)*, KPCPb^–/–^shControl* (*n* = 3), and *KPCPb^–/–^shVimentin* (*n* = 3) PDAC cells. *P < 0.05, Student *t* test. Data shown as mean ± SE. (**H**) Quantification of CK19-positive liver metastasis in mice treated with simvastatin (*n* = 3), Withaferin A (*n* = 3), and each vehicle control (*n* = 3) with splenic injection of *KPC and KPCPb^–/–^* PDAC cells, determined by combining 3 independent sections. *P < 0.05, Student *t* test. Data are represented as mean ± SE. (**I**) Representative IHC analysis of PBRM1 and vimentin in human PDACs. Patient #1 shows high PBRM1 expression and low expression of vimentin. Patient #2 shows the low expression of PBRM1 and the high expression of vimentin. Scale bar: 50 μm. Data are representative of 3 independent experiments. (**J**) Analysis of high vimentin expression in human PDACs (*n* = 105) surgically resected with high (*n* = 50) or low (*n* = 55) PBRM1 expression as determined by IHC. *P < 0.05, Pearson’s χ^2^ test.

Given that vimentin is a critical factor for cell migration, metastasis, and EMT (33, 34), we next focused on vimentin among the 25 genes. We hypothesized that vimentin inhibition would reverse the high tumor-grade phenotype and decrease metastasis of *Pbrm1*-null PDAC in mice. To test this hypothesis, we silenced the vimentin gene expression by using short hairpin RNA (shRNA) in control *KPC* and *KPCPb^–/–^* PDAC cells. Quantitative real-time PCR analyses revealed no significant change in the expression of *Snai1*, *Snai2*, *Zeb1*, *Twist1, Cldn7*, and *Dsg2* with the knockdown of vimentin in control *KPC* PDAC cells (Figure 7A). Quantitative real-time PCR analysis of *Vimentin*-silenced *KPCPb^–/–^* PDAC cells (*KPCPb^–/–^shVimentin*) confirmed reduced expression of the vimentin gene and the EMT-related gene *Snai1* and increased expression of apical junction genes, including *Cldn7* and *Dsg2* (Figure 7B). Next, we subcutaneously allografted *KPC* and *KPCPb^–/–^* mouse PDAC cells with shRNA knockdown of the vimentin gene. We found almost no histological changes in subcutaneous tumors with the knockdown of vimentin in *KPC* PDAC cells (Figure 7C). Notably, vimentin inhibition in *KPCPb^–/–^* PDAC cells reversed the tumor grade of PDAC from poorly differentiated to well differentiated, and the loss of vimentin expression was confirmed by IHC analysis (Figure 7C and Supplemental Figure 7A). Splenic injections were performed using *KPC* and *KPCPb^–/–^* mouse PDAC cells with shRNA knockdown of the vimentin gene. The splenic injection model using *KPC* PDAC cells showed no significant changes in the rate of the CK19-positive area in the liver with the knockdown of vimentin. In contrast, the splenic injection model using *KPCPb^–/–^* mouse PDAC cells with shRNA knockdown of the vimentin gene showed a reduced metastatic tumor burden compared with *KPCPb^–/–^* mouse PDAC cells with control shRNA (Figure 7, D and E). Notably, almost all the liver metastatic lesions formed in mice injected with vimentin-silenced *KPCPb^–/–^* PDAC cells were composed of vimentin-positive PDAC cells (Supplemental Figure 7B), indicating these lesions originated from vimentin-positive PDAC cells that had escaped vimentin silencing. Consistent with the in vivo data, the scratch assay showed no significant changes with the knockdown of vimentin in a *Pbrm1*-proficient context, whereas *KPCPb^–/–^* mouse PDAC cells with shRNA knockdown of the vimentin exhibited reduced migratory activity compared with control cells (Figure 7, F and G). These data indicate that *Pbrm1*-null PDAC is specifically dependent on vimentin in progression in contrast to *Pbrm1*-WT PDAC.

Next, we performed experiments with the knockdown of other important EMT players, including *Snai1, Snai2*, and *Twist1* in both *Pbrm1*-deficient and -proficient contexts. As a result, almost no histological changes were observed with the knockdown of *Snai1, Snai2*, and *Twist1*, in both *Pbrm1*-deficient and -proficient contexts (Supplemental Figure 7, C and D). In addition, RT-PCR showed no significant changes in the expression of *Snai1*, *Snai2*, *Zeb1*, *Twist1, Cldn7*, and *Dsg2* with the knockdown of *Snai1, Snai2*, and *Twist1* in both *Pbrm1*-deficient and -proficient contexts except for the expression of each knocked-down gene, and *Cldn7* and *Dsg2* that was upregulated with the knockdown of *Snai2* in a *Pbrm1-*deficient context, and *Snai2* that was downregulated with the knockdown of *Twist1* in a *Pbrm1-*proficient context (Supplemental Figure 7E). Invasion assay showed no significant changes with the knockdown of *Snai1, Snai2*, and *Twist1* in both *Pbrm1*-deficient and -proficient contexts (Supplemental Figure 8A). The splenic injection model showed no significant changes in the rate of the CK19-positive area in the liver with the knockdown of *Snai1, Snai2*, and *Twist1* in both *Pbrm1*-deficient and -proficient contexts (Supplemental Figure 8B). Therefore, we conclude that the reverse of the phenotype in *Pbrm1-*null PDAC is exclusively attributed to the knockdown of vimentin, but not in general to other players of EMT.

To determine whether *Pbrm1*-null PDAC cells were sensitive to treatment with vimentin inhibitors, we next performed treatment with vimentin inhibitors, simvastatin (35), and Withaferin A (36), in the splenic injection mouse model. We found that liver metastases were reduced in the splenic injection model of *Pbrm1*-null PDAC cells by administration of vimentin inhibitors, indicating that *Pbrm1*-deficient aggressive PDAC was sensitive to treatment with vimentin inhibitors (Figure 7H). Vimentin immunostaining showed vimentin reorganization and perinuclear bundling or perinuclear remaining, as shown in the previous reports (Supplemental Figure 8C) (35, 36), indicating that vimentin inhibitors sufficiently worked in the mice. These results indicate that vimentin inhibition in *Pbrm1*-null PDAC cells reversed the tumor grade from poorly to well-differentiated PDAC, thereby reducing the metastasis of *Pbrm1*-null PDAC in mice. Therefore, PBRM1 is a critical determinant of tumor grade, EMT, and metastasis in PDAC and functions by directly regulating vimentin expression.

### PBRM1 expression correlates with vimentin expression in human PDACs.

Eventually, we determined whether the PBRM1/vimentin axis existed in human PDACs. We performed IHC analysis for PBRM1 and vimentin using serial sections from 105 human PDAC tissue samples (Figure 7I). Seventy-six percent of human PDAC samples with low levels of PBRM1 expression displayed high levels of vimentin expression, whereas only 32% of the samples with high levels of PBRM1 expression displayed high levels of vimentin expression. Low PBRM1 expression significantly correlated with high vimentin expression in human PDACs (Figure 7J and Supplemental Figure 8D). Furthermore, an inquiry into the TCGA-PAAD cohort revealed that high mRNA expression of the vimentin gene correlated with the basal-like molecular subtype rather than the classical subtype in human PDACs (Supplemental Figure 8E). Collectively, consistent with the mouse data, these results indicate that low PBRM1 expression correlates with more aggressive characteristics, including high vimentin gene expression, poorly differentiated PDAC, and basal molecular subtype in human PDACs, demonstrating the clinical relevance of these findings in mice.

## Discussion

In this study, we demonstrated for what we believe to be the first time that PBRM1 is a critical determinant of tumor grade, EMT, and metastasis in PDAC and functions by directly downregulating vimentin expression. First, we demonstrated that pancreatic *Pbrm1* deletion resulted in the accelerated formation of poorly differentiated PDAC, concomitant with increased metastasis and worse prognosis in mice with activated *Kras* and heterozygous or homozygous disruption of *Trp53*, which established that PBRM1 plays tumor-suppressive roles in PDAC formation and progression. Second, we demonstrated that *Pbrm1* ablation in established PDAC converted the tumor grade from well-differentiated to poorly differentiated PDAC or undifferentiated pancreatic carcinoma in vivo. Third, *Pbrm1*-null PDAC exhibited decreased expression of apical junction genes and increased expression of EMT pathway genes, including vimentin, and also typical markers of the squamous subtype of p63 and ΔNp63. Fourth, vimentin inhibition in *Pbrm1*-null PDAC cells reversed the dedifferentiation phenotype, thereby decreasing EMT and metastasis in mice. Mechanistically, we revealed that PBRM1 binds to the promoter region of the vimentin gene and directly downregulates its expression in PDAC cells. Fifth, consistent with the mouse data, low expression of PBRM1 was associated with high vimentin expression, poorly differentiated PDAC, postoperative recurrence, and poor prognosis in human PDACs. In addition, TCGA data analysis revealed that PDAC with *PBRM1* deletion was significantly correlated with a worse tumor grade and an aggressive squamous molecular subtype. These data highlight the clinical relevance of our findings.

Metastasis is the primary cause of death in more than 90% of patients with cancer (37). EMT is associated with tumor metastasis and progression (38). Vimentin is one of the most commonly used markers of mesenchymal features (39), and its expression has been reported to increase metastatic potential (38). Notably, the expression of known EMT regulators, including vimentin, was upregulated in PDAC cells from *KPCPb^–/–^* mice. We demonstrated that pancreatic *PBRM1* deletion significantly increased vimentin expression in PDAC cells and induced subsequent EMT, resulting in increased metastasis. The expression of vimentin is reported to be regulated by several transcription factors, including positive-acting factors such as activator protein 1 (AP-1) (40), nuclear factor-kappa B (NF-kB) (41, 42), and Stat3 (43), as well as negative regulators such as ZNF148 (44). In this study, we demonstrate that the chromatin regulator PBRM1 binds to the vimentin promoter region and epigenetically regulates its expression in pancreatic cancer.

Our data revealed that *Pbrm1* loss synergized with oncogenic *Kras* and heterozygous or homozygous *Trp53* deletion to form poorly differentiated PDAC or undifferentiated pancreatic carcinoma with squamous subtype properties. The expression of genes upregulated in the squamous molecular subtype of human PDAC was also upregulated in PDAC from *KPCPb^–/–^* mice. Additionally, our IHC analysis of PDAC from *KPC* and *KPCPb^–/–^* mice revealed that the expression of ΔNp63 and p63, which are typical master regulators of the squamous phenotype, was upregulated in PDAC from *KPCPb^–/–^* mice. Consistently, human PDAC with *PBRM1* deletion exhibited a squamous molecular subtype. IHC analysis of 12 human pancreatic squamous cell carcinoma or adenosquamous carcinoma samples revealed that the expression of ΔNp63 was upregulated in all the samples and that PBRM1 expression was lost in 83% of those.

Regarding the relationship between PBRM1 and p63, our RNA-seq and ChIP-seq data suggested that PBRM1 did not directly regulate p63 expression (data not shown); however, the expression of vimentin and p63 has been reported to be correlated in esophageal squamous carcinoma cells (45), suggesting a possible indirect relation between PBRM1 and p63 mediated by vimentin.

We demonstrated that PBRM1 played a critical role in both PDAC initiation and progression. During tumor progression, PBRM1 directly regulates vimentin expression, and PBRM1 deletion results in the activation of the EMT and subsequent metastasis. However, there may be a downstream target of PBRM1, other than vimentin, involved in PDAC initiation, and the role of vimentin in PDAC initiation remains to be verified.

The SWI/SNF complex is classified into PBAF, cBAF, and ncBAF. PBRM1 and ARID1A are specific subunits in PBAF and cBAF, respectively. SMARCB1 is a shared subunit in cBAF and PBAF. Interestingly, each component of the SWI/SNF complex has a different function in pancreatic cancer. Loss of ARID1A promotes an EMT phenotype and MYC-facilitated protein synthesis in PDAC cells (46, 47). Depletion of *Smarcb1* activates the Myc signaling, driving an anabolic synthesis that increases protein metabolism and activates adaptive ER-stress–induced survival pathways (48). SMARCD3 is amplified and enriched in pancreatic cancer stem cells. SMARCD3 cooperates with FOXA1 to control lipid and fatty acid metabolism, resulting in therapy resistance and poor prognosis in cancer (49). Brg1 regulates the hypoxia pathway, strongly affecting cell survival, stem-like property, and metastasis of PDAC (50). The previous reports, together with this study, indicate that PBRM1, ARID1A, and SMARCB1 play tumor-suppressive roles and inhibit EMT in pancreatic cancer. BRG1 and SMARCD3, which are shared subunits in cBAF, PBAF, and ncBAF, play oncogenic roles and are important for stemness in pancreatic cancer (49, 50). Therefore, it is considered that PBAF and cBAF have tumor-suppressive roles and are important for epithelial differentiation in pancreatic cancer, and ncBAF has an oncogenic role that overwhelms the tumor-suppressive role of PBAF and cBAF. Further study is needed to unravel the precise function of cBAF, PBAF, and ncBAF.

In this study, we demonstrated that vimentin inhibition in *Pbrm1*-null PDAC nullified the dedifferentiation phenotype induced by PBRM1 deletion, thereby decreasing metastasis in mice. These data suggest that the PBRM1/vimentin axis could be a potential therapeutic target for human PDAC. Further investigations are needed to determine whether vimentin inhibitors (51) have therapeutic effects in patients with PDAC with low PBRM1 expression, poorly differentiated PDAC, squamous molecular subtype PDAC, or undifferentiated pancreatic carcinoma.

In conclusion, we demonstrated that PBRM1 is a critical determinant of tumor grade and metastasis in PDAC and functions by directly regulating vimentin expression. Consistently, low PBRM1 expression in human PDACs correlates with high vimentin expression, poorly differentiated PDAC, high recurrence rate, and reduced overall survival, emphasizing the clinical relevance of the mouse findings. Further understanding of the mechanisms of cancer differentiation and metastasis will lead to the development of therapeutic strategies for pancreatic cancer.

## Methods

### Sex as a biological variable.

Sex was not considered as a biological variable. Our study examined males and females, and similar findings were reported for both sexes.

### Mice.

The following mouse strains were used: *Ptf1a^Cre^* (24) (a gift from Yoshiya Kawaguchi, Kyoto University), *Ptf1a^CreER^* (52), *LSL-Kras^G12D^* (53) (a gift from David Tuveson, Cold Spring Harbor Laboratory, Cold Spring Harbor, New York, USA), *Trp53^flox^* (Stock no. 008462; The Jackson Laboratory, Bar Harbor, Maine, USA), *Pbrm1^f/f^* (Stock no. 029049; The Jackson Laboratory), *Pdx1-Flp*, *FSF-Kras^G12D^*, and *FSF-R26^CAG–CreERT2^* (28). Mice were crossed in a mixed background and no selection for a specific sex was performed in this study. Tamoxifen (Sigma-Aldrich) was dissolved in corn oil and administered intraperitoneally at a concentration of 2 mg/mouse for 5 days.

### Clinical samples.

A total of 117 surgically resected PDAC tissue specimens were obtained from patients admitted to the Kyoto University Hospital.

### Histology and IHC analysis.

For histological analyses, mouse pancreata and liver were formalin fixed, embedded in paraffin, and sectioned at a thickness of 5 μm using a Leica RM2165 microtome. Paraffin-embedded sections were stained with H&E. Pancreatic lesions were classified using the same diagnostic criteria as those used in humans and were reviewed by a pathologist. For IHC analysis, antigen retrieval was performed by boiling the sections in 10 mM citric acid buffer (pH 6.0) or EDTA buffer (pH 8.0) for 15 minutes at 98°C. The samples were then incubated with primary antibodies (Fibronection [Abcam, ab2413, 1:100, RRID:AB_2262874], RFP [ROCKLAND, 600-401-379, 1:500, ROCKLAND, 600-401-379], Vimentin [CST, #5741S, 1:200, RRID:AB_10695459], CK19 [Abcam, ab52625, 1:200, RRID:AB_2281020, Sigma-Aldrich, MABT913, 1:200, RRID:AB_2892523], Amylase [Abcam, ab21156, 1:300, RRID:AB_446061], PBRM1 [Bethyl, A301-591A, 1:500, RRID:AB_1078808], p63 [Abcam, ab124762, 1:300, RRID:AB_10971840], ΔNp63 [Abcam, ab203826, 1:200, RRID:AB_2934266]) overnight at 4°C or for 2 hours at 23°C in a humidified chamber, followed by secondary antibody incubation for one hour at 23°C. Secondary antibodies used were as follows: goat anti-rabbit (Vector Laboratories, BA-1000, 1:200, RRID:AB_2313606), donkey anti-rabbit, Alexa Fluor488 (Abcam, ab150073, 1:200, RRID:AB_2636877), donkey anti-goat, Alexa Fluor 555 (Abcam, ab150130, RRID:AB_2927775), and donkey anti-rat, Alexa Fluor 555 (Abcam, ab150154, RRID:AB_2813834). For immunofluorescence staining, sections were counterstained with Hoechst 33342 (Thermo Fisher Scientific). Peroxidase-streptavidin labeling was performed using a VECTASTAIN Elite ABC Standard Kit (Cat. no. PK-6100; Vector Laboratories). The sections were then stained with a diaminobenzidine substrate (Cat. no. K3468; Dako) and counterstained with hematoxylin (Cat. no. 109249; Sigma-Aldrich). For quantitative analysis of PanIN areas, PDAC or undifferentiated carcinoma areas, metastatic areas, or pancreatic acinar cell areas, the Alcian blue-positive PanIN areas, CK19-positive metastasis areas, or amylase-positive acinar cell areas were measured using ImageJ software (National Institutes of Health). Three whole pancreatic sections from each mouse were analyzed. Whole pancreatic areas were measured using a BZ-X Analyzer (Keyence). Two blinded investigators conducted an IHC analysis to evaluate the intensity of PBRM1 and vimentin expression in human PDAC tissues and divided them into high- and low-expression groups. We analyzed 3 independent sections and evaluated the staining intensity of the cancer cells in four grades: none, weak, moderate, and strong. If more than half of the cancer cells showed none or weak intensity, we assigned the sample to a low-expression group. If not, we assigned the sample to the high-expression group. Homogenized stool samples were centrifuged, and the supernatant was stained with Oil Red O solution (Sigma-Aldrich).

### Cell culture of primary mouse PDAC cell lines.

Primary pancreatic tumors resected from the mice were minced using scissors. After digestion with 2.5 mg/mL collagenase D (Roche) at 37°C for 15 minutes with agitation, the tissue fragments were further dissociated using a gentle MACS dissociator (Miltenyi Biotec). The cells were passed through a 100 μm cell strainer and plated on a dish in DMEM containing 10% FBS and 50 U/mL penicillin-streptomycin. Cultures were maintained at 37°C in 5% CO_2_.

### Extended methods.

Additional details on compounds, reagents, assays, and bioinformatics analysis are described in the Supplemental Methods.

### Statistics.

Data are shown as mean ± SE. All statistical analyses were performed using R (version 4.3.1). 2-tailed Student *t* tests were employed for comparisons. If the data did not meet this test, a Mann-Whitney U- test was used. Paired *t* tests were employed for analyzing the difference between pairs of measurements. Contingency tables were analyzed using Pearson’s χ^2^ tests. If the expected values in any of the cells of a contingency table were below 5, 2-tailed Fisher’s exact tests were used with Bonferroni correction for multiple testing. Statistical significance between groups of 3 or more was determined by a 1-way ANOVA, followed by Tukey’s multiple comparison test.

Survival was measured by the Kaplan-Meier method and analyzed by Log-rank (Mantel-Cox) test. Cox proportional hazard analysis was employed to estimate univariate and multivariate hazard ratio and 95% CI. Values of *P* < 0.05 were considered as significantly different. All in vitro experiments, except the ChIP-Seq and the primary acinar cell isolation, were performed with at least 3 biological replicates.

### Study approval.

All mouse experiments were approved by the IACUC and the Ethics Committee of Kyoto University. Patient samples were used only for staining. The Ethics Committee of Kyoto University approved the use of patient samples for this experiment without requiring written informed consent. Informed consent was obtained through an opt-out on the website. The study conformed to the provisions of the Declaration of Helsinki. The study protocol (R2904) was approved by the Ethics Committee of Kyoto University.

### Data availability.

The RNA-seq data generated in this study have been deposited in GenBank under accession number PRJNA1241453. The ChIp-seq raw data generated in this study have been deposited in GenBank under accession number PRJNA1241467. Microscopy data reported in this paper will be shared upon request. Values for all data points in graphs are reported in the Supporting Data Values file.

## Author contributions

MK and AF conceived and designed the study. T Masui and EH provided clinical samples. MK, T Maruno, MI, K Iimori, KM, K Iwane, GY, MO, M Namikawa, MS, T Masuda, YF, M Nagao, OA, and TY acquired data. MK, AF, SO, YH, MT, T Maruno, YN, DS, TT, HS interpreted data. MK and AF wrote the manuscript, and MK, AF, and HS revised it.

## Supplementary Material

Supplemental data

Supporting data values

## Figures and Tables

**Table 1:**
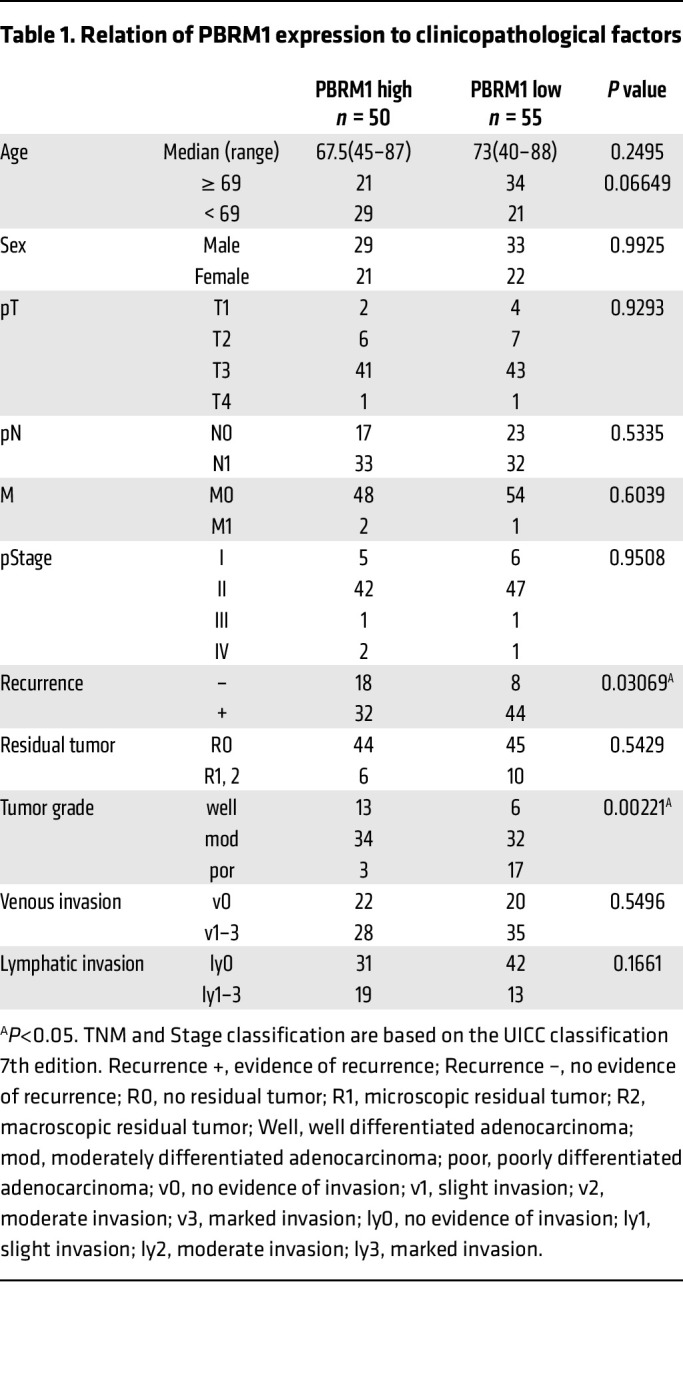
Relation of PBRM1 expression to clinicopathological factors

## Version 1 06/02/2025

Electronic publication
