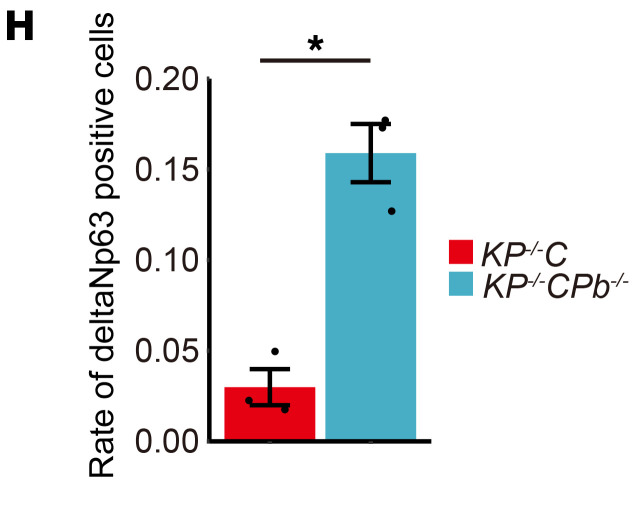# Corrigendum to Polybromo 1/vimentin axis dictates tumor grade, epithelial-mesenchymal transition, and metastasis in pancreatic cancer

**DOI:** 10.1172/JCI206371

**Published:** 2026-04-01

**Authors:** Munenori Kawai, Akihisa Fukuda, Munehiro Ikeda, Kei Iimori, Kenta Mizukoshi, Kosuke Iwane, Go Yamakawa, Mayuki Omatsu, Mio Namikawa, Makoto Sono, Tomonori Masuda, Yuichi Fukunaga, Munemasa Nagao, Osamu Araki, Takaaki Yoshikawa, Satoshi Ogawa, Yukiko Hiramatsu, Motoyuki Tsuda, Takahisa Maruno, Yuki Nakanishi, Dieter Saur, Tatsuaki Tsuruyama, Toshihiko Masui, Etsuro Hatano, Hiroshi Seno

Original citation: *J Clin Invest*. 2025;135(11):e177533. https://doi.org/10.1172/JCI177533

Citation for this corrigendum: *J Clin Invest*. 2026;136(7):e206371. https://doi.org/10.1172/JCI206371

After publication of their article, the authors noticed that the labels “*KP^–/–^C*” and “*KP^–/–^CPb^–/–^*” in Figure 4H were inadvertently switched. The corrected figure panel is shown below. The HTML and PDF versions of the paper have been updated.

The authors regret the error.

## Figures and Tables

**Figure:**