# Supplementary material for: Polybromo 1/vimentin axis dictates tumor grade, epithelial-mesenchymal transition, and metastasis in pancreatic cancer
Source: J Clin Invest. 2025 Jun 2;135(11):e177533. doi: 10.1172/JCI177533 (PMC12126225; doi:10.1172/JCI177533)
Supplement: Supplemental data [file jci-135-177533-s284.pdf]

Supplementary methods

Acinar Cell Isolation

Mouse pancreata were minced into small pieces and digested with Collagenase P (0.4 mg/mL) at 37°C for 10 minutes. After two washes with Hanks' Balanced Salt solution buffer containing 5% fetal bovine serum, the tissue suspension was filtered through a 100-μm cell strainer. The flow-through was layered onto Hanks' Balanced Salt solution plus 30% fetal bovine serum solution and centrifuged. Subsequently, the cell pellet was used for total RNA extraction.

RNA Isolation and Quantitative Reverse Transcription Polymerase Chain Reaction

Total RNA was extracted from the cells using an RNA mini Kit (Qiagen, Hilden, Germany). Single-stranded complementary DNA (cDNA) was synthesized using the ReverTra Ace qPCR RT Kit (TOYOBO, Tokyo, Japan). qRT-PCR was performed using SYBR Green Master Mix (Roche) and a LightCycler 480 (Roche). The expression levels were standardized by comparing them to the levels of beta-actin. The primer sequences are listed in Supplementary Table 4.

RNA-seq and Analysis

Total RNA extracted from PDAC cell lines using the RNA mini Kit (Qiagen, Hilden, Germany) was used for RNA-seq, which was performed by Macrogen, Inc. (Seoul, South Korea) on the Novaseq6000 platform with 2 × 100 bp paired-end sequencing using the SMART-Seq v4 Ultra Low Input RNA kit and TruSeq RNA Sample Prep Kit v2. Adaptors and low-quality bases were trimmed from the reads using Trimmomatic (version 0.39)(1) with the default parameters. Reads were mapped to the Mus musculus reference genome build mm10 using STAR (version 2.7.3a),(2) and counted using RSEM (version v1.3.1)(3). The read count data were normalized using the iDEGES/edgeR method. Normalized count data were used for GSEA(4), and differentially expressed genes were determined using Tag Count Comparison (TCC)(5) with a P value < 0.05.

Chromatin Immunoprecipitation

ChIP assay was performed using the SimpleChIP® Plus Enzymatic Chromatin IP Kit (Cell Signaling cat # 9005) according to the manufacturer's protocol. Briefly, isolated PDAC cells were cross-linked with 1% formaldehyde and incubated for 10 min with gentle shaking at room temperature. Crosslinking was stopped by

adding glycine for 5 min with gentle shaking. After washing with phosphate-buffered saline supplemented with protease inhibitors, the fixed cells were lysed. Nuclei were pelleted, digested with 0.5 μl micrococcal nuclease for 20 minutes at 37 °C to fragment chromatin, resuspended in Chip buffer (provided in the kit), and briefly sonicated. Digested chromatin (1/50) was kept aside as input, and the remaining chromatin was immunoprecipitated with 10 μg rabbit IgG antibody (provided in the kit), 2 μg anti-PBRM1 antibody (Bethyl, A301-591A), and 2 μg anti-H3K27ac antibody (Abcam, ab4729). The chromatin was collected using magnetic beads and decross-linked. DNA was purified using DNA spin columns. The purified DNA was submitted to Macrogen (Japan) for library preparation and sequencing. Sequencing libraries were prepared using the TruSeq ChIP Sample Prep Kit (Illumina) according to the manufacturer’s protocol and sequenced using 151-bp paired-end reads on the NovaSeq 6000 sequencing platform.

ChIP-Seq data analysis

Reads were aligned to the mouse (mm10) genome using Bowtie2(6) (version 2.4.2) with default parameters, and only uniquely aligned reads were retained using Samtools (version 1.11). MACS2 (version 2.2.7.1) was used for peak

calling to identify the chromatin domains enriched for PBRM1 binding and histone modification marks. The GREAT (version 4.0.4) online tool was used to identify genes linked to promoters or enhancers based on the following association rules: basal plus extension:5 kb upstream, 1 kb downstream, and 1000 kb max extension. Bigwig files for browser-track visualization were generated using DeepTools (version. 3.5.0) and visualized using Integrative Genomics Viewer (IGV) (version 2.8.12).

Lentivirus Transduction and Infection of Pancreatic Cancer Cells

Silencing of *Vim*, *Snai1*, *Snai2*, and *Twist1* was achieved using pLKO-shVim (Vim MISSION shRNA SHCLND-NM_011701 TRCN0000089828), pLKO-shSnai1 (Snai1 MISSION shRNA SHCLND-NM_011427 TRCN0000096620), pLKO-shSnai2 (Snai2 MISSION shRNA SHCLND-NM_011415 TRCN0000096226), and pLKO-shTwist1 (Twist1 MISSION shRNA SHCLND-NM_011658 TRCN0000095077) for *KPC* and *KPCPb*$^{-/-}$ cells. To produce lentiviruses, HEK293T cells were transfected with the plasmids, pCAG-HIVgp, and pCMV-VSV-G-RSV-Rev (gifts from Dr. Hiroyuki Miyoshi, RIKEN BioResource Center, Tsukuba, Japan). Culture supernatants were collected 48 h after transfection,

filtered, and concentrated using PEG-it (System Biosciences, Palo Alto, CA, USA; catalog no. LV810A-1) and resuspended in Hanks' balanced salt solution. Infection was performed overnight in the presence of polybrene (8 μg/mL), followed by selection using puromycin.

Xenografted tumor model

For orthotopic injection, *KPC* and *KPCPb*$^{-/-}$ tumor cells (5.0×10^5) in 50 μL medium were injected into the pancreas of six- to eight-week-old C57BL/6 mice. The mice were then subjected to analysis after three weeks.

For subcutaneous injection, *KPC* and *KPCPb*$^{-/-}$ tumor cells (5.0×10^5) in 50 μL medium were injected into the flank of the six- to eight-week-old C57BL/6 mice. The mice were then subjected to analysis after three weeks.

In vitro wound-healing assays

Wound-healing assays were evaluated using *KPC* and *KPCPb*$^{-/-}$ tumor cells. Cells were seeded on plates and grown into a monolayer. Serum starvation was performed to minimize proliferation by switching to a serum-free medium once a monolayer was formed. A 200 μl pipette tip was used to scratch the monolayer.

After 24 h, the wound closure rate was quantified in eight high-power fields (HPFs) using ImageJ software.

The experimental model of liver metastases

Ten-week-old C57BL/6 mice were anesthetized with isoflurane. Tumor cells (2 × 10^5) derived from *KPC* and *KPCPb*$^{-/-}$ mice were suspended in DMEM/10% FBS. The cells were injected into mouse spleens using a 27G syringe during open laparotomy. Liver metastases were analyzed after 10 days.

Pharmacological assay

Gemcitabine (MedChemExpress) was dissolved in phosphate-buffered saline and administered intraperitoneally at a concentration of 50 mg/kg(7) 3 times a week to *KP*$^{-/-}$*C* and *KP*$^{-/-}$*CPb*$^{-/-}$ mice from the age of 4 weeks.

For splenic injection, simvastatin (Tokyo Chemical Industry) was dissolved in phosphate-buffered saline and administered intraperitoneally at a concentration of 5 mg/kg(8) once every 2 days after injection of cancer cells to mouse spleen. Liver metastases were analyzed after 10 days.

For subcutaneous injection, simvastatin was dissolved in phosphate-buffered saline and administered intraperitoneally at a concentration of 5 mg/kg once every 2 days, 10 days after injection of cancer cells. The mice were subjected to analysis three weeks after injection of cancer cells.

For splenic injection, Withaferin A (AdipoGen Life Sciences) was dissolved in 5% DMSO and 95% corn oil and administered intraperitoneally at a concentration of 2 mg/kg(9) once every 2 days after injection of cancer cells to mouse spleen. Liver metastases were analyzed after 10 days.

For subcutaneous injection, Withaferin A was dissolved in 5% DMSO and 95% corn oil and administered intraperitoneally at a concentration of 2 mg/kg once every 2 days, 10 days after injection of cancer cells. The mice were subjected to analysis three weeks after injection of cancer cells.

The Cancer Genome Atlas Data Set Analysis

The Cancer Genome Atlas (Firehose legacy) data of 147 patients with pancreatic cancer in terms of mutations, copy number alterations, and messenger RNA expression were obtained from cBioPortal(10, 11). Cases with shallow deletion

or deep deletion were considered *PBRM1* deletions by analyzing the *PBRM1* copy number alterations.

1. Bolger AM, Lohse M, Usadel B. Trimmomatic: A flexible trimmer for Illumina sequence data. *Bioinformatics*. 2014;30(15):2114–2120.

2. Dobin A, et al. STAR: Ultrafast universal RNA-seq aligner. *Bioinformatics*. 2013;29(1):15–21.

3. Li B, Dewey CN. RSEM: Accurate transcript quantification from RNA-Seq data with or without a reference genome. *BMC Bioinformatics*. 2011;12. https://doi.org/10.1186/1471-2105-12-323.

4. Subramanian A, et al. *Gene set enrichment analysis: A knowledge-based approach for interpreting genome-wide expression profiles*. 2005.

5. Sun J, et al. TCC: An R package for comparing tag count data with robust normalization strategies. *BMC Bioinformatics*. 2013;14(1). https://doi.org/10.1186/1471-2105-14-219.

6. Langmead B, Salzberg SL. Fast gapped-read alignment with Bowtie 2. *Nat Methods*. 2012;9(4):357–359.

7. Zheng X, et al. Epithelial-to-mesenchymal transition is dispensable for metastasis but induces chemoresistance in pancreatic cancer. *Nature*. 2015;527(7579):525–530.

8. Zhao Y, et al. Simvastatin Inhibits Inflammation in Ischemia-Reperfusion Injury. *Inflammation*. 2014;37(5):1865–1875.

9. Thaiparambil JT, et al. Withaferin A inhibits breast cancer invasion and metastasis at sub-cytotoxic doses by inducing vimentin disassembly and serine 56 phosphorylation. *Int J Cancer*. 2011;129(11):2744–2755.

10. Gao J, et al. *integrative analysis of complex cancer genomics and clinical Profiles Using the cBioPortal inTRODUcTiOn eQUiPMenT inSTRUcTiOnS Querying individual cancer Studies Viewing and interpreting the Results Performing cross-cancer Queries Viewing cancer Study Summary Data Viewing genomic alterations in a Single Tumor: Patient View Programmatic access nOTeS anD ReMaRKS complementary Data Sources and analysis Options Future Directions*. 2013.

11. Cerami E, et al. The cBio Cancer Genomics Portal: An open platform for exploring multidimensional cancer genomics data. *Cancer Discov*. 2012;2(5):401–404.

Supplementary Figure Legends

Supplementary Figure 1

A Representative immunohistochemical (IHC) analysis of high (n = 50) and low (n = 55) PBRM1 expression in human pancreatic ductal adenocarcinoma samples. Scale bar, 50 μm.

B GSEA enrichment plots of the MOFFITT BASAL LIKE, COLLISSON QUASI-MESENCHYMAL, and PID_DELTA_NP63_PATHWAY gene sets. NES, normalized enrichment score; FDR, false discovery rate.

C The rate of the patients classified to *PBRM1* deletion (n = 42) or *PBRM1* diploid (n = 102) in basal like (n = 61), squamous (n = 28), or quasi-mesenchymal (n = 31) subtype in the TCGA-PAAD cohort. Pearson's chi-square test.

D The mRNA expression of *CKS1B, HSPE1, EIF2S2, KIF18A, UBE2C, CKAP2L, and CCNA2* in *PBRM1* diploid PDAC (n = 101) and PDAC with *PBRM1* deletion (n = 42) from a cohort of 143 patients in the TCGA dataset. *$P < 0.05$, Mann-Whitney U- test.

E The upper table shows analysis of deltaNp63 expression in human pancreatic adenosquamous carcinoma (n = 11) and squamous cell carcinoma (n = 1) with high (n = 2) or low (n = 10) PBRM1 expression as determined by immunohistochemistry. The lower table shows analysis of deltaNp63 expression in human PDAC with high (n = 11) or low (n = 13) PBRM1 expression as determined by immunohistochemistry.

F Representative PBRM1 staining in human undifferentiated carcinoma samples (n = 6). Scale bar, 50 µm.

G Rates of high and low PBRM1 IHC levels in human well-and moderately differentiated PDAC (well+mod) (n = 85) and undifferentiated carcinoma (undif) (n = 6). Fisher's exact tests.

Supplementary Figure 2

A Representative immunohistochemistry of PBRM1 in murine pancreatic samples, including normal pancreas, premalignant PanIN lesions, and pancreatic ductal adenocarcinomas (PDAC) derived from *Ptf1a*^{Cre}*; LSL-Kras*^{G12D} (*KC*) and *Ptf1a*^{Cre}*; LSL-Kras*^{G12D}*; Trp53*$^{f/wt}$ (*KPC*) mice. PanIN, pancreatic intraepithelial

neoplasia. Scale bar, 50 μm. Data are representative of 3 independent experiments.

B Relative pancreas-to-body weight ratio in *Ptf1a*^{Cre} (*C*) (n = 3) and *Ptf1a*^{Cre}*; Pbrm1*$^{f/f}$ (*CPb*$^{-/-}$) (n = 3) mice at six weeks of age. Data are represented as mean ± SEM. Student *t*-test.

C Representative H&E, PBRM1, Amylase, and CK19 staining in *CPb*$^{-/-}$, *Ptf1a*^{Cre}*; Pbrm1*$^{f/wt}$ (*CPb*$^{+/-}$), and *C* mice at six weeks of age. Scale bar, 50 μm. Data are representative of 3 independent experiments.

D Relative *Pbrm1* mRNA expression in acinar cells isolated from *CPb*$^{-/-}$ (n = 2), *CPb*$^{+/-}$ (n = 2), and *C* (n = 1) mice.

Supplementary Figure 3

A Representative H&E staining of *KC*, *Ptf1a*^{Cre}*; LSL-Kras*^{G12D}*; Pbrm1*$^{f/wt}$(*KCPb*$^{+/-}$), and *Ptf1a*^{Cre}*; LSL-Kras*^{G12D}*; Pbrm1*$^{f/f}$ (*KCPb*$^{-/-}$) mice at four, eight, and 12 weeks old. Scale bar, 50 μm. Data are representative of 3 independent experiments.

B Analysis for regions of ADM and PanIN1/2/3 in *KC* (n = 3), *KCPb*$^{+/-}$ (n = 3), and *KCPb*$^{-/-}$ (n = 3) mice pancreata.

C Kaplan-Meier plots showing the overall survival in the cohorts of *KC* (n = 86), $KCPb^{+/-}$ (n = 41), and $KCPb^{-/-}$ (n = 54) mice.

D Representative oil red O staining of stool collected from *KC* and $KCPb^{-/-}$ mice at the age of 20 weeks. Data are representative of 3 independent experiments.

E Scatterplot and regression line of pancreas/body weight ratio in relation to age of *KC* (n = 57) and $KCPb^{-/-}$ (n = 31) mice.

F Representative H&E staining of each invasive carcinoma in *KC*, $KCPb^{+/-}$, and $KCPb^{-/-}$ mice at the moribund state. Data are representative of 3 independent experiments.

G Representative coimmunostaining of CK19 and vimentin in PDAC from *KC* (n = 3), $KCPb^{+/-}$ (n = 3), and $KCPb^{-/-}$ (n = 3) mice. Scale bar, 50 μm.

H Quantification of the rate of the vimentin-positive cancer cells in PDAC from *KC* (n = 3), $KCPb^{+/-}$ (n = 3), and $KCPb^{-/-}$ (n = 3) mice. **P* < 0.05, Student *t*-test. Data shown as mean ± SE.

Supplementary Figure 4

A Representative CK19 and Pbrm1 staining in PDAC from $Ptf1a^{Cre}$; $LSL\text{-}Kras^{G12D}$; $Trp53^{f/wt}$ (*KPC*), $Ptf1a^{Cre}$; $LSL\text{-}Kras^{G12D}$; $Trp53^{f/wt}$; $Pbrm1^{f/wt}$ ($KPCPb^{+/-}$)

and *Ptf1a*^{Cre}*; LSL-Kras*^{G12D}*; Trp53*$^{f/wt}$*; Pbrm1*$^{f/f}$ (*KPCPb*$^{-/-}$) mice at the primary site. Scale bar, 50 μm. The bottom left image is also shown in Supplementary Figure 2A. Data are representative of 3 independent experiments.

B Representative H&E staining in undifferentiated carcinoma from *KPCPb*$^{-/-}$ mice, showing spindle and multinuclear cells with scant cytoplasm, indistinct cell borders, and hyperchromatic nuclei. Scale bar, 50 μm. Data are representative of 3 independent experiments.

C Analysis for regions of well, moderately, and poorly differentiated PDAC and undifferentiated carcinoma in *KPC* (n = 3), *KPCPb*$^{+/-}$ (n = 3) and *KPCPb*$^{-/-}$ (n = 3) mice pancreata at the moribund state.

D Representative CK19 and PBRM1 staining in the livers of *KPC* mice and metastatic PDAC in the livers of *KPCPb*$^{+/-}$ and *KPCPb*$^{-/-}$ mice. Scale bar, 50 μm. Data are representative of 3 independent experiments.

E Representative p63 staining in PDAC from *KPC* and *KPCPb*$^{-/-}$ mice at the primary site. Scale bar, 50 μm. Data are representative of 3 independent experiments.

F Representative images of PDAC cells from *KPC* and *KPCPb*$^{-/-}$ mice in cell culture dishes. Data are representative of 3 independent experiments.

Supplementary Figure 5

A The genetic strategy used to activate oncogenic *Kras,* and delete *Pbrm1* and *Trp53* specifically in the pancreas from the embryonic stage.

B Representative H&E staining of the pancreas from $Ptf1a^{Cre}$; $LSL\text{-}Kras^{G12D}$; $Trp53^{f/f}$ ($KP^{-/-}C$), $Ptf1a^{Cre}$; $LSL\text{-}Kras^{G12D}$; $Trp53^{f/f}$; $Pbrm1^{f/wt}$ ($KP^{-/-}CPb^{+/-}$), and $Ptf1a^{Cre}$; $LSL\text{-}Kras^{G12D}$; $Trp53^{f/f}$; $Pbrm1^{f/f}$ ($KP^{-/-}CPb^{-/-}$) mice at five weeks of age. Scale bar, 50 μm. Data are representative of 3 independent experiments.

C Representative H&E staining with whole pancreas in $Ptf1a^{Cre}$; $LSL\text{-}Kras^{G12D}$; $Trp53^{f/f}$ (*KP-/-C*), $Ptf1a^{Cre}$; $LSL\text{-}Kras^{G12D}$; $Trp53^{f/f}$; $Pbrm1^{f/wt}$($KP^{-/-}CPb^{+/-}$), and $Ptf1a^{Cre}$; $LSL\text{-}Kras^{G12D}$; $Trp53^{f/f}$; $Pbrm1^{f/f}$ ($KP^{-/-}CPb^{-/-}$) mice at three and five weeks of age. Data are representative of 3 independent experiments.

D Representative amylase staining of the whole pancreas in $KP^{-/-}C$ and $KP^{-/-}CPb^{-/-}$ mice at three and five weeks of age. Data are representative of 3 independent experiments.

E Quantification of amylase-positive pancreatic acinar cell areas in $KP^{-/-}C$ (n = 3) and $KP^{-/-}CPb^{-/-}$ (n = 3) mice at three and five weeks of age. *$P < 0.05$, Student *t*-test. Data represent mean ± SE.

F Representative H&E staining of *KP$^{-/-}$C* mice in a moribund state with normal acinar cell components. Scale bar, 500 μm. Data are representative of 3 independent experiments.

G The genetic strategy used to activate oncogenic *Kras,* delete *Pbrm1* and *Trp53*, and express tdTomato specifically in the pancreas from the embryonic stage.

H Representative coimmunostaining of Fibronectin, and tdTomato in PDAC from *Ptf1a^{Cre}; LSL-Kras^{G12D} Trp53$^{f/f}$*; *LSL-Rosa$^{td\text{-}tomato}$* (*KP$^{-/-}$CTomato*) (n = 3) and *Ptf1a^{Cre}; LSL-Kras^{G12D}; Trp53$^{f/f}$; Pbrm1$^{f/f}$*; *LSL-Rosa$^{td\text{-}tomato}$* (*KP$^{-/-}$CPb$^{-/-}$Tomato*) (n = 3) mice. Scale bar, 50 μm.

Supplementary Figure 6

A The genetic strategy used to activate oncogenic *KRAS,* and delete *PBRM1* and *Trp53* specifically in adult acinar cells upon tamoxifen treatment.

B Representative H&E staining of PDAC at the primary site and liver, and representative PBRM1 and vimentin staining at the primary site from *Ptf1a^{CreER}; LSL-Kras^{G12D}; Trp53$^{f/wt}$* (*ER-KPC*), *Ptf1a^{CreER}; LSL-Kras^{G12D}; Trp53$^{f/wt}$; Pbrm1$^{f/wt}$* (*ER-KPCPb$^{+/-}$*), *Ptf1a^{CreER}; LSL-Kras^{G12D}; Trp53$^{f/wt}$; Pbrm1$^{f/f}$* (*ER-KPCPb$^{-/-}$*) mice. Scale bar, 50 μm. Data are representative of 3 independent experiments.

C ChIP data of the PBRM1 binding region in the vimentin gene promoter and coding regions in the RH-4 rhabdomyosarcoma cell line. TSS: transcription start site.

Supplementary Figure 7

A Representative CK19, and vimentin staining in PDAC allografted subcutaneously with *KPCPb$^{-/-}$shVimentin* and *KPCPb$^{-/-}$shcontrol*. Scale bar, 50 µm. Data are representative of 3 independent experiments.

B Representative vimentin staining in metastatic PDAC after injection into the spleen with *KPCPb$^{-/-}$shControl*, and *KPCPb$^{-/-}$shVimentin* PDAC cells. Scale bar, 500 µm. Data are representative of 3 independent experiments.

C Representative H&E staining in PDACs allografted subcutaneously with *KPCshSnai1*, *KPCshSnai2,* and *KPCshTwist1* PDAC cells. Scale bar, 50 µm. Data are representative of 3 independent experiments.

D Representative H&E staining in PDACs allografted subcutaneously with *KPCPb$^{-/-}$shSnai1*, *KPCPb$^{-/-}$shSnai2,* and *KPCPb$^{-/-}$shTwist1* PDAC cells. Scale bar, 50 µm. Data are representative of 3 independent experiments.

E Quantitative real-time PCR analysis of the relative mRNA expression of *Zeb1*, *Vim*, *Snai1, Snai2, Twist1, Cldn7,* and *Dsg2* in *KPCshSnai1* (n = 3), *KPCshSnai2* (n = 3)*, KPCshTwist1* (n = 3)*, KPCPb$^{-/-}$shSnai1* (n = 3), *KPCPb$^{-/-}$shSnai2* (n = 3)*,* and *KPCPb$^{-/-}$shTwist1* (n = 3) PDAC cells compared to each *shControl* (n = 3). **P* < 0.05, Student *t*-test. Data shown as mean ± SE.

Supplementary Figure 8

A Quantification of the scratch assay with *KPC shControl* (n = 3), *KPCshVimentin* (n = 3)*, KPCshSnai1* (n = 3), *KPCshSnai2* (n = 3), *KPCshTwist1* (n = 3), *KPCPb$^{-/-}$shControl* (n = 3), *KPCPb$^{-/-}$shVimentin* (n = 3), *KPCPb$^{-/-}$shSnai1* (n = 3), *KPCPb$^{-/-}$shSnai2* (n = 3), and *KPCPb$^{-/-}$shTwist1* (n = 3) PDAC cells. **P* < 0.05, Student *t*-test. Data shown as mean ± SE.

B Quantification of CK19-positive liver metastasis with a splenic injection of *KPC shControl* (n = 3), *KPCshVimentin* (n = 3)*, KPCshSnai1* (n = 3), *KPCshSnai2* (n = 3), *KPCshTwist1* (n = 3), *KPCPb$^{-/-}$shControl* (n = 3), *KPCPb$^{-/-}$shVimentin* (n = 3), *KPCPb$^{-/-}$shSnai1* (n = 3), *KPCPb$^{-/-}$shSnai2* (n = 3), and *KPCPb$^{-/-}$shTwist1* (n = 3) PDAC cells, as determined by combining three independent sections. **P* < 0.05, Student *t*-test. Data are represented as mean ± SE.

C Representative coimmunostaining of Vimentin, and CK19 in PDAC allografted subcutaneously with *KPCPb*$^{-/-}$ and *KPC* PDAC cells in mice treated with simvastatin, Withaferin A, and each vehicle control. Scale bar, 50 μm. Data are representative of 3 independent experiments.

D Analysis of vimentin expression in surgically resected human PDACs (n = 105) with high (n = 50) or low (n = 55) PBRM1 expression as determined by immunohistochemistry.

E Analysis of molecular subtyping in human PDACs with high (n = 73) and low (n = 74) vimentin expression using the TCGA dataset (n = 147). $^{*}P < 0.05$, Pearson's chi-square test.

Supplementary Table 1. Univariate analysis for overall survival in pancreatic cancer.

		HR (95% CI)	*p* value
Age	69 <=	1.382 (0.903-2.115)	0.136
	69 >	1	
Sex	Male	1.269 (0.824-1.95)	0.281
	Female	1	
Tumor grade	well/mod	1	0.000921*
	por	2.389 (1.427-3.999)	
pT	T1, 2	1	0.906
	T3, 4	1.034 (0.5922-1.806)	
pN	N0	1	0.000701*
	N1	2.22 (1.40-3.52)	
pM	M0	1	0.508
	M1	1.477 (0.4652-4.692)	
v	v0	1	0.142
	v1-3	1.393 (0.895-2.17)	
ly	ly0	1	0.0032*
	ly1-3	1.979 (1.257-3.115)	
Residual tumor	R0	1	0.000052*
	R1, 2	3.216 (1.826-5.662)	
Recurrence	-	1	0.000000129*
	+	6.941 (3.381-14.25)	
PBRM1	Low	1.735 (1.13-2.663)	0.0118*
	High	1	

*p<0.05

HR, hazard ratio; 95% CI, 95% confidence interval; well, well differentiated adenocarcinoma; mod, moderately differentiated adeno- carcinoma; poor, poorly differentiated adenocarcinoma.

Supplementary Table 2. Multivariate analysis for overall survival in pancreatic cancer.

		HR (95% CI)	*p* value
Age	69 <=	1.3798 (0.8459-2.251)	0.1972
	69 >		
Sex	Male	1.1525 (0.7252-1.832)	0.5483
	Female	1	
Tumor grade	well/mod	1	
	por	1.1421 (0.6151-2.120)	0.67395
pT	T1, 2	1	
	T3, 4	0.9005 (0.4469-1.814)	0.76938
pN	N0	1	
	N1	1.5460 (0.8676-2.755)	0.13939
pM	M0	1	
	M1	1.4384 (0.4034-5.129)	0.57517
v	v0	1	
	v1-3	0.9702 (0.5587-1.685)	0.91446
ly	ly0	1	
	ly1-3	2.1804 (1.2784-3.719)	0.00422*
Residual tumor	R0	1	
	R1	1.9969 (1.0209-3.906)	0.04335*
Recurrence	-	1	
	+	6.1437 (2.7418-13.766)	0.0000103*
PBRM1	Low	1.7498 (1.0421-2.938)	0.03435*
	High	1	

*p<0.05

HR, hazard ratio; 95% CI, 95% confidence interval; well, well differentiated adenocarcinoma; mod, moderately differentiated adeno- carcinoma; poor, poorly differentiated adenocarcinoma.

Supplementary Table 3. Genes directly regulated by Pbrm1 with modifications of H3K27ac status.

Downregulated by Pbrm1	Upregulated by Pbrm1
Acvr2a	5730507C01Rik
Bpgm	Abcc2
Cdkn2c	Abcc3
Cyp39a1	Ank2
Dennd5b	Anxa11
Dnaaf2	Arhgef3
Elmo1	Ccdc125
Fmnl3	Cdx2
Fyco1	Cgnl1
Hipk2	Cltc
Hsph1	Col14a1
Jade1	Cp
Klhl42	Cst6
Lztfl1	Cuedc1
Mapt	Dsp
Mindy3	Egfr
Mis18bp1	Eps8l3
Nudt5	F3
P3h2	Fasn
Pet117	Fer1l6
Ppp2r3a	Fgd6
Stk17b	Flywch2
Syne3	Gipc2
Uhrf2	Gm14399
Vim	Gprc5b
	Hmga2
	Igf1
	Igsf5
	Il17rd
	Itga2
	Itpr1
	Kank1
	Kdm6b
	Lama2
	Lama5
	Lamc2
	Lilr4b
	Lrig1
	Maml2
	Mapk6
	Mmp14
	Mtcl1
	Nes
	Pdgfb

Pkdcc
Prss22
Rab31
Rps6ka2
Serinc5
Shroom2
Sik2
Smad3
Sort1
Sox9
St3gal1
St8sia6
Syn3
Taok3
Tbkbp1
Tgfa
Tmtc2
Tnip1
Tspan9
Vps37b
Wnt7b
Zfp422
Zfp931
Zfp947

Supplementary Table 4. Primer Sets for Quantitative Reverse Transcription Polymerase Chain Reaction Analyses

Gene symbol	Forward	Reverse
mouse		
Pbrm1	AAGATGCCCTTGTACTGCATAAA	AGTCGCCGATACCGATTACTT
Cdh1	CTCCAGTCATAGGGAGCTGTC	TCTTCTGAGACCTGGGTACAC
Cdh2	AGGCTTCTGGTGAAATTGCAT	GTCCACCTTGAAATCTGCTGG
Cldn4	AGCCCTTATGGTCATCAGCATC	ATGCTTGCCACGATGAACAC
Cldn7	CTTGACGCCCATGAACGTTAAG	ACGCAGCTTTGCTTTCACTG
Dsc2	TTCCCGGGCATCAAAACAAC	AGTTGTCAAGCCGTTTGTGG
Dsg2	AATGAAGGCAAACCGTTCCC	TGGCAATCGGGTTCTTTCTG
Snai1	CACACGCTGCCTTGTGTCT	GGTCAGCAAAAGCACGGTT
Snai2	TGGTCAAGAAACATTTCAACGCC	GGTGAGGATCTCTGGTTTTGGTA
Twist1	GGACAAGCTGAGCAAGATTCA	GAGCCCTCTCCTAAGAGACCC
Vim	TCCACACGCACCTACAGTCT	CCGAGGACCGGGTCACATA
Zeb1	GCTGGCAAGACAACGTGAAAG	GCCTCAGGATAAATGACGGC

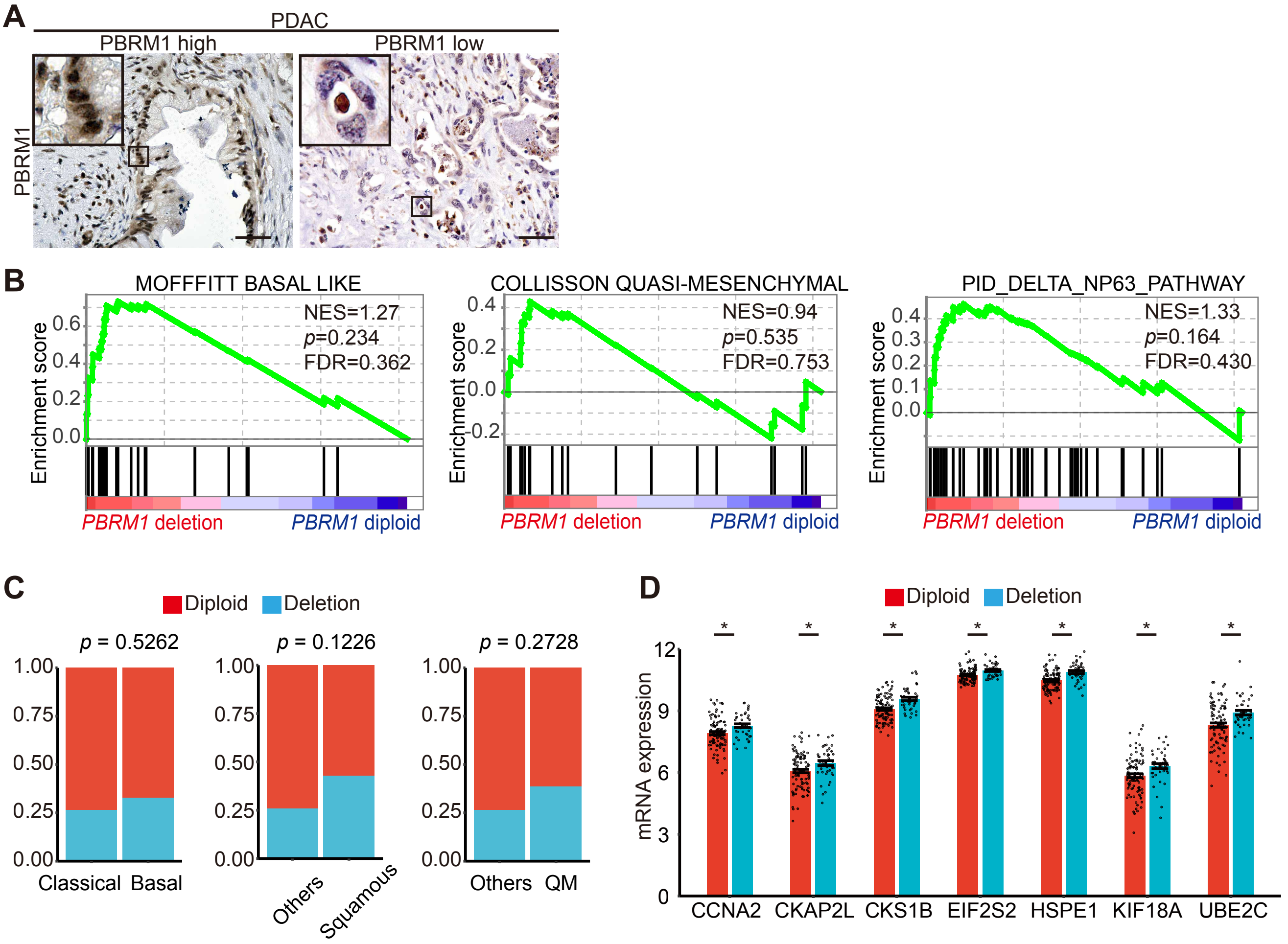

E

Adenosquamous carcinoma + squamous cell carcinoma

	PBRM1 high	PBRM1 low
deltaNp63 posi	2	10
deltaNp63 neg	0	0

PDAC

	PBRM1 high	PBRM1 low
deltaNp63 posi	0	1
deltaNp63 neg	11	12

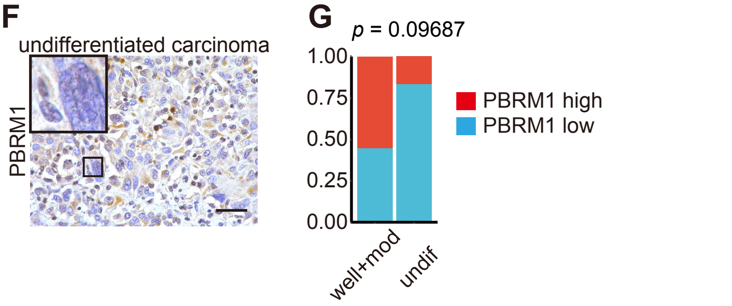

Supplementary Figure 1

A Representative immunohistochemical (IHC) analysis of high (n = 50) and low (n = 55) PBRM1 expression in human pancreatic ductal adenocarcinoma samples. Scale bar, 50 µm.

B GSEA enrichment plots of the MOFFITT BASAL LIKE, COLLISSON QUASI-MESENCHYMAL, and PID_DELTA_NP63_PATHWAY gene sets. NES, normalized enrichment score; FDR, false discovery rate.

C The rate of the patients classified to PBRM1 deletion (n = 42) or PBRM1 diploid (n = 102) in basal like (n = 61), squamous (n = 28), or quasi-mesenchymal (n = 31) subtype in the TCGA-PAAD cohort. Pearson's chi-square test.

D The mRNA expression of CKS1B, HSPE1, EIF2S2, KIF18A, UBE2C, CKAP2L, and CCNA2 in *PBRM1* diploid PDAC (n = 101) and PDAC with *PBRM1* deletion (n = 42) from a cohort of 143 patients in the TCGA dataset. *P < 0.05, Mann-Whitney U- test.

E The upper table shows analysis of deltaNp63 expression in human pancreatic adenosquamous carcinoma (n = 11) and squamous cell carcinoma (n = 1) with high (n = 2) or low (n = 10) PBRM1 expression as determined by immunohistochemistry. The lower table shows analysis of deltaNp63 expression in human PDAC with high (n = 11) or low (n = 13) PBRM1 expression as determined by immunohistochemistry.

F Representative PBRM1 staining in human undifferentiated carcinoma samples (n = 6). Scale bar, 50 µm.

G Rates of high and low PBRM1 IHC levels in human well-and moderately differentiated PDAC (well+mod) (n = 85) and undifferentiated carcinoma (undif) (n = 6). Fisher's exact test.

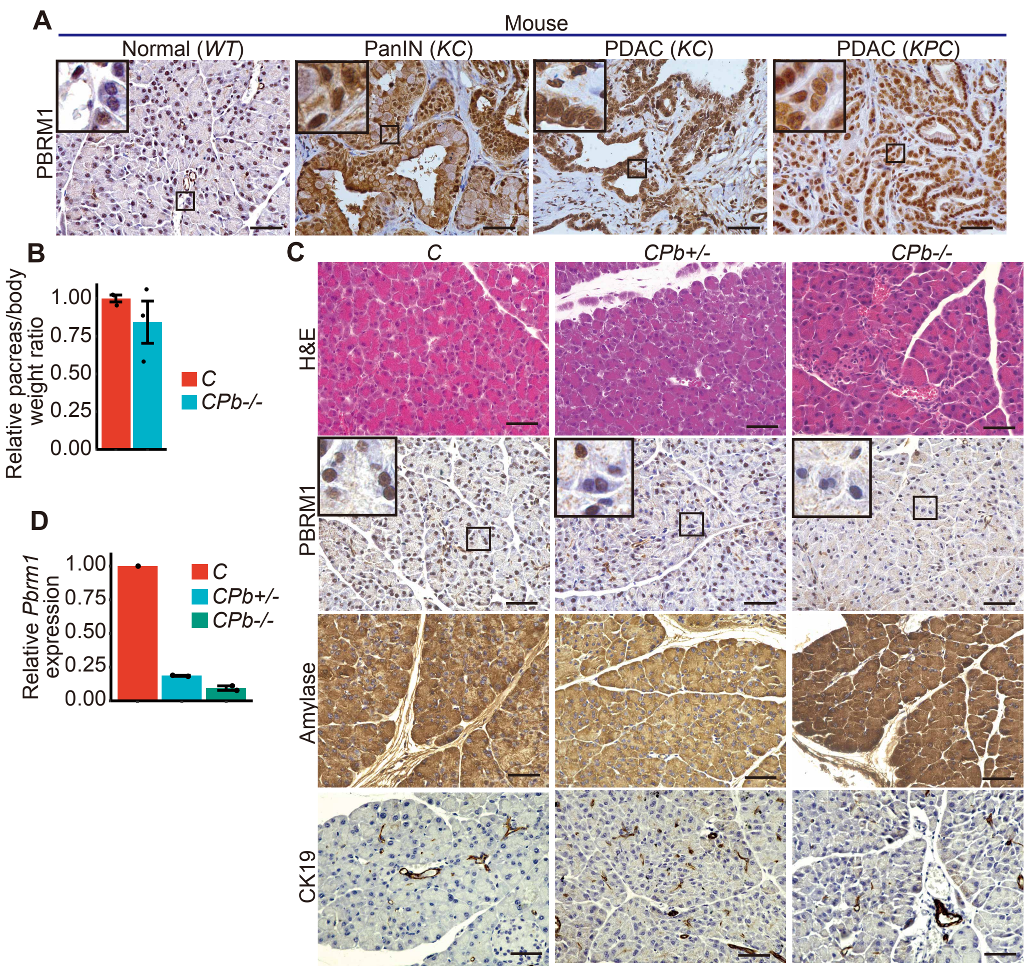

Supplementary Figure 2

A Representative immunohistochemistry of PBRM1 in murine pancreatic samples, including normal pancreas, premalignant PanIN lesions, and pancreatic ductal adenocarcinomas (PDAC) derived from *Ptf1a*^{Cre}; *LSL-Kras*^{G12D}(*KC*) and *Ptf1a*^{Cre}; *LSL-Kras*^{G12D}; *Trp53*$^{f/wt}$ (*KPC*) mice. PanIN, pancreatic intraepithelial neoplasia. Scale bar, 50 µm. Data are representative of 3 independent experiments.
B Relative pancreas-to-body weight ratio in *Ptf1a*^{Cre} (*C*) (n = 3) and *Ptf1a*^{Cre}; *Pbrm1*$^{f/f}$ (*CPb*$^{-/-}$) (n = 3) mice at six weeks of age. Data are represented as mean ± SEM. Student *t*-test.
C Representative H&E, PBRM1, Amylase, and CK19 staining in *CPb*$^{-/-}$, *Ptf1a*^{Cre}; *Pbrm1*$^{f/wt}$ (*CPb*$^{+/-}$), and *C* mice at six weeks of age. Scale bar, 50 µm. Data are representative of 3 independent experiments.
D Relative *Pbrm1* mRNA expression in acinar cells isolated from *CPb*$^{-/-}$ (n = 2), *CPb*$^{+/-}$ (n = 2), and *C* (n = 1) mice.

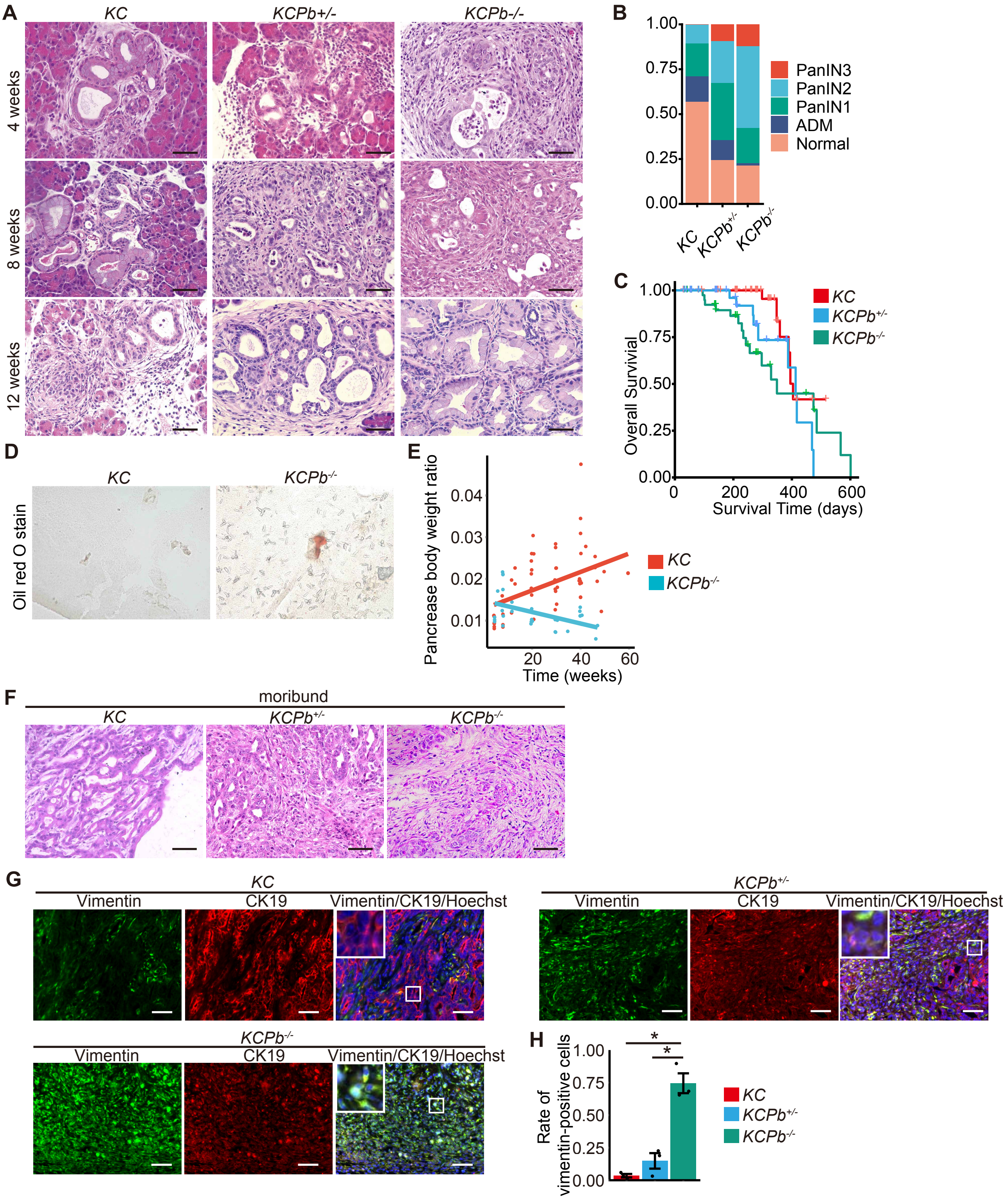

Supplementary Figure 3

A Representative H&E staining of *KC*, *Ptf1a*^{Cre}; *LSL-Kras*^{G12D}; *Pbrm1*$^{f/wt}$ (*KCPb*$^{+/-}$), and *Ptf1a*^{Cre}; *LSL-Kras*^{G12D}; *Pbrm1*$^{f/f}$ (*KCPb*$^{-/-}$) mice at four, eight, and 12 weeks old. Scale bar, 50 µm. Data are representative of 3 independent experiments.
B Analysis for regions of ADM and PanIN1/2/3 in *KC* (n = 3), *KCPb*$^{+/-}$ (n = 3), and *KCPb*$^{-/-}$ (n = 3) mice pancreata
C Kaplan-Meier plots showing the overall survival in the cohorts of *KC* (n = 86), *KCPb*$^{+/-}$ (n = 41), and *KCPb*$^{-/-}$ (n = 54) mice.
D Representative oil red O staining of stool collected from *KC* and *KCPb*$^{-/-}$ mice at the age of 20 weeks. Data are representative of 3 independent experiments.
E Scatterplot and regression line of pancreas/body weight ratio in relation to age of *KC* (n = 57) and *KCPb*$^{-/-}$ (n = 31) mice.
F Representative H&E staining of each invasive carcinoma in *KC, KCPb*$^{+/-}$, and *KCPb*$^{-/-}$ mice at the moribund state. Scale bar, 50 µm. Data are representative of 3 independent experiments.
G Representative coimmunostaining of CK19 and vimentin in PDAC from *KC* (n = 3), *KCPb*$^{+/-}$ (n = 3), and *KCPb*$^{-/-}$ (n = 3) mice at the moribund state. Scale bar, 50 µm.
H Quantification of the rate of the vimentin-positive cancer cells in PDAC from *KC* (n = 3), *KCPb*$^{+/-}$ (n = 3), and *KCPb*$^{-/-}$ (n = 3) mice. *$P < 0.05$, Student *t*-test. Data shown as mean ± SE.

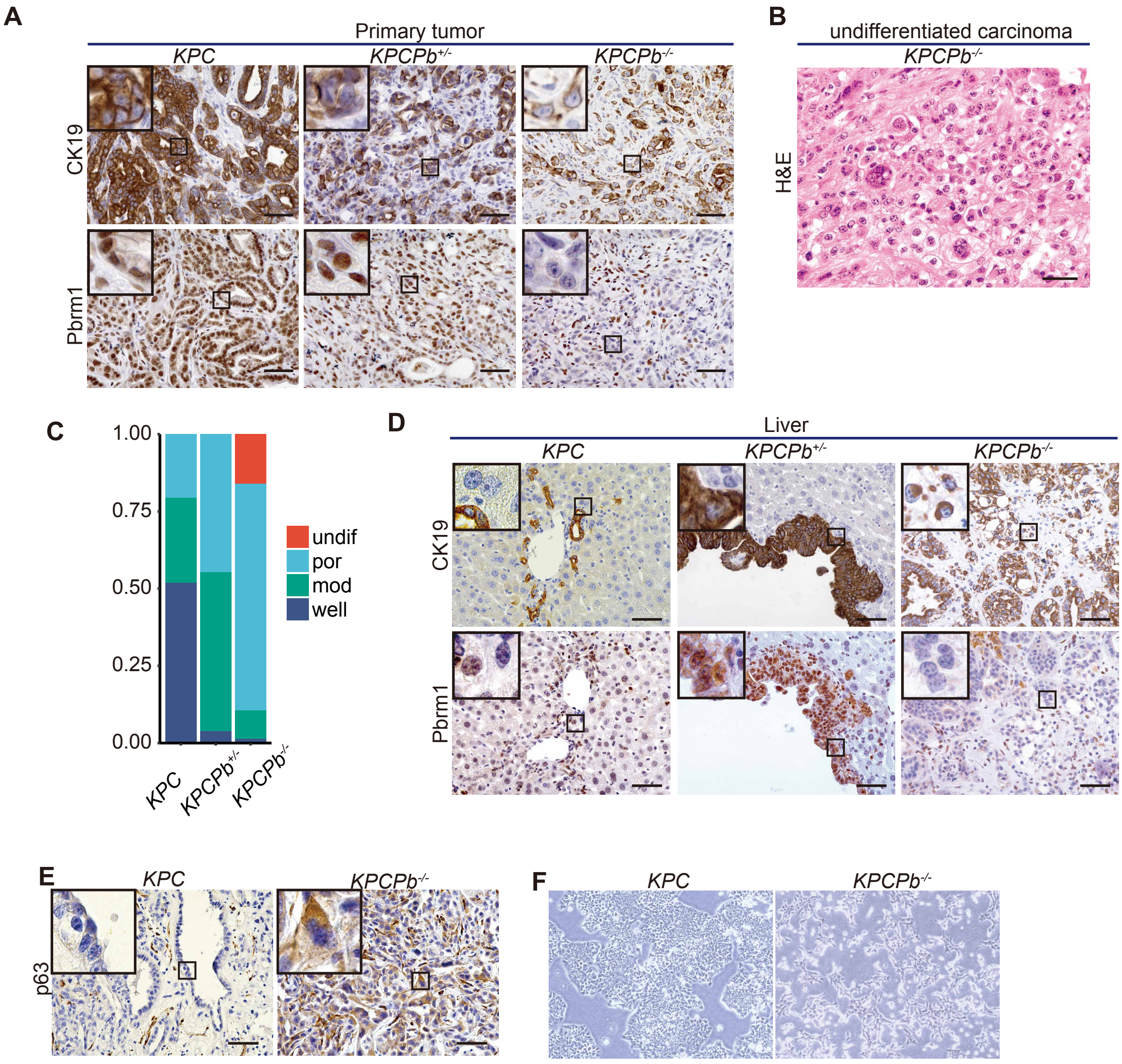

Supplementary Figure 4

A Representative CK19 and Pbrm1 staining in PDAC from *Ptf1a*^{Cre}; *LSL-Kras*^{G12D}; *Trp53*$^{f/wt}$ (*KPC*), *Ptf1a*^{Cre}; *LSL-Kras*^{G12D}; *Trp53*$^{f/wt}$; *Pbrm1*$^{f/wt}$ (*KPCPb*$^{+/-}$), and *Ptf1a*^{Cre}; *LSL-Kras*^{G12D}; *Trp53*$^{f/wt}$; *Pbrm1*$^{f/f}$ (*KPCPb*$^{-/-}$) mice at the primary site. Scale bar, 50 μm. The bottom left image is also shown in Supplementary Figure 2A. Data are representative of 3 independent experiments.
B Representative H&E staining in undifferentiated carcinoma from *KPCPb*$^{-/-}$ mice, showing spindle and multinuclear cells with scant cytoplasm, indistinct cell borders, and hyperchromatic nuclei. Scale bar, 50 μm. Data are representative of 3 independent experiments.
C Analysis for regions of well, moderately, and poorly differentiated PDAC and undifferentiated carcinoma in *KPC* (n = 3), *KPCPb*$^{+/-}$ (n = 3) and *KPCPb*$^{-/-}$ (n = 3) mice pancreata at the moribund state.
D Representative CK19 and PBRM1 staining in the livers of *KPC* mice and metastatic PDAC in the livers of *KPCPb*$^{+/-}$ and *KPCPb*$^{-/-}$ mice. Scale bar, 50 μm. Data are representative of 3 independent experiments.
E Representative p63 staining in PDAC from *KPC* and *KPCPb*$^{-/-}$ mice at the primary site. Scale bar, 50 μm. Data are representative of 3 independent experiments.
F Representative images of PDAC cells from *KPC* and *KPCPb*$^{-/-}$ mice in cell culture dishes. Data are representative of 3 independent experiments.

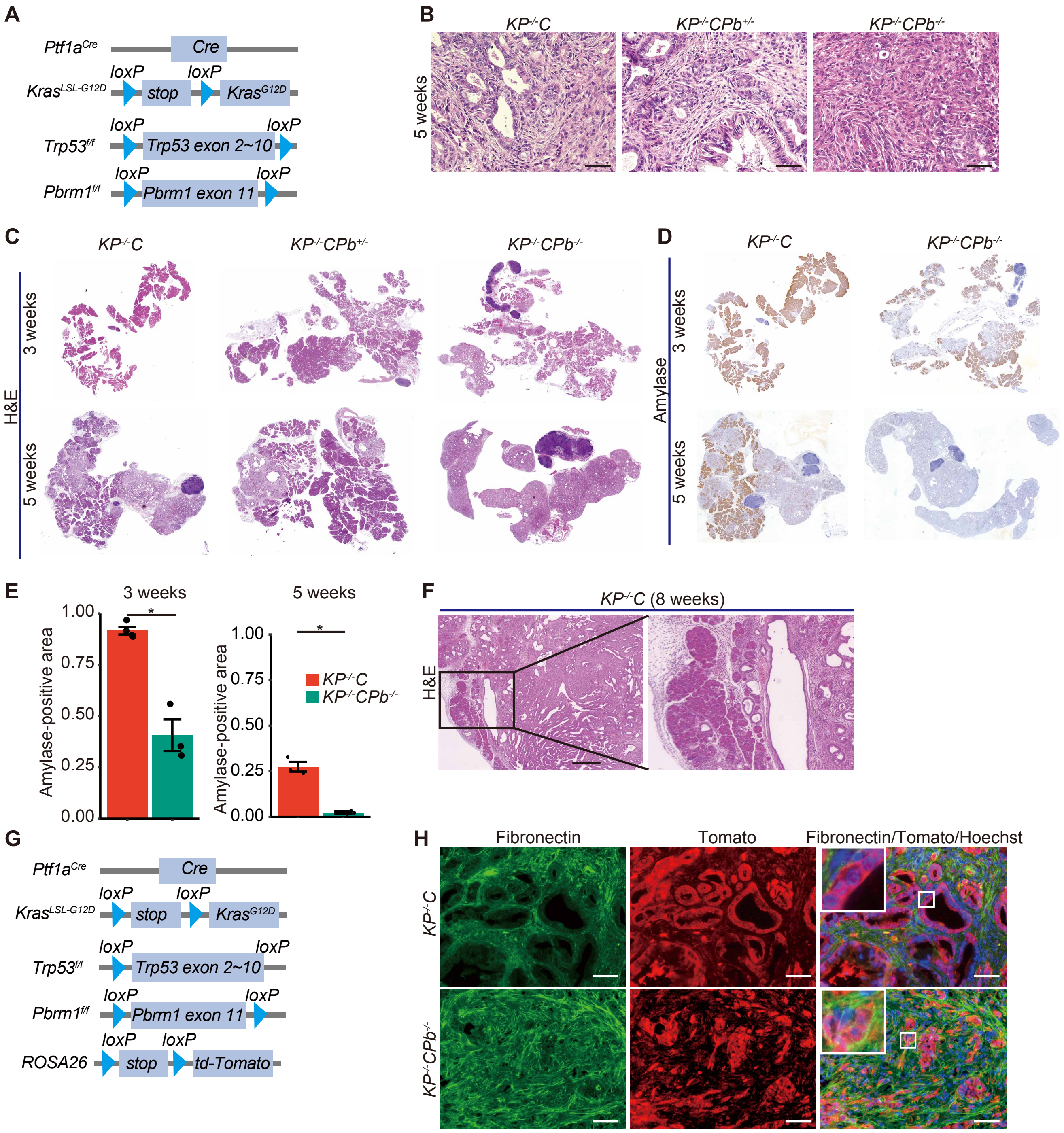

Supplementary Figure 5

A The genetic strategy used to activate oncogenic *Kras*, and delete *Pbrm1* and *Trp53* specifically in the pancreas from the embryonic stage.

B Representative H&E staining of the pancreas from *Ptf1a*^{Cre}; *LSL-Kras*^{G12D}; *Trp53*$^{f/f}$ (*KP*$^{-/-}$*C*), *Ptf1a*^{Cre}; *LSL-Kras*^{G12D}; *Trp53*$^{f/f}$; *Pbrm1*$^{f/wt}$ (*KP*$^{-/-}$*CPb*$^{+/-}$), and *Ptf1a*^{Cre}; *LSL-Kras*^{G12D}; *Trp53*$^{f/f}$; *Pbrm1*$^{f/f}$ (*KP*$^{-/-}$*CPb*$^{-/-}$) mice at five weeks of age. Scale bar, 50 μm. Data are representative of 3 independent experiments.

C Representative H&E staining with whole pancreas in *KP*$^{-/-}$*C*, *KP*$^{-/-}$*CPb*$^{+/-}$, and *KP*$^{-/-}$*CPb*$^{-/-}$ mice at three and five weeks of age. Data are representative of 3 independent experiments.

D Representative amylase staining of the whole pancreas in *KP*$^{-/-}$*C* and *KP*$^{-/-}$*CPb*$^{-/-}$ mice at three and five weeks of age. Data are representative of 3 independent experiments.

E Quantification of amylase-positive pancreatic acinar cell areas in *KP*$^{-/-}$*C* (n = 3) and *KP*$^{-/-}$*CPb*$^{-/-}$ (n = 3) mice at three and five weeks of age. *$P < 0.05$, Student *t*-test. Data represent mean ± SE.

F Representative H&E staining of *KP*$^{-/-}$*C* mice in a moribund state with normal acinar cell components. Scale bar, 500 μm. Data are representative of 3 independent experiments.

G The genetic strategy used to activate oncogenic *Kras*, and delete *Pbrm1* and *Trp53*, and express tdTomato specifically in the pancreas from the embryonic stage.

H Representative coimmunostaining of Fibronectin, and tdTomato in PDAC from *Ptf1a*^{Cre}; *LSL-Kras*^{G12D}; *Trp53*$^{f/f}$; *LSL-Rosa26*^{tdTomato} (*KP*$^{-/-}$*CTomato*) (n = 3) and *Ptf1a*^{Cre}; *LSL-Kras*^{G12D}; *Trp53*$^{f/f}$; *Pbrm1*$^{f/f}$; *LSL-Rosa26*^{tdTomato} (*KP*$^{-/-}$*CPb*$^{-/-}$*Tomato*) (n = 3) mice. Scale bar, 50 μm.

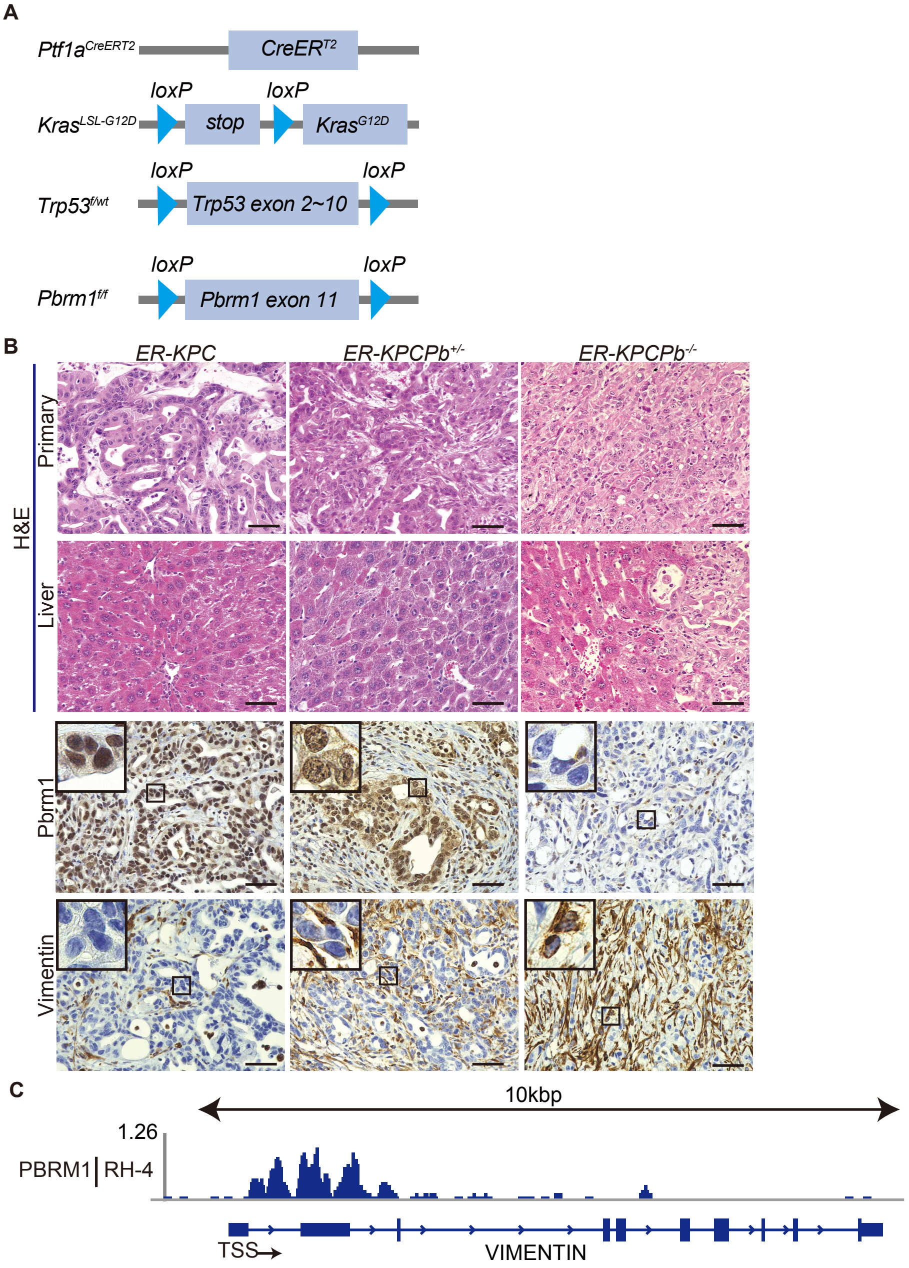

Supplementary Figure 6

A The genetic strategy used to activate oncogenic *KRAS*, and delete *PBRM1* and *Trp53* specifically in adult acinar cells upon tamoxifen treatment.

B Representative H&E staining of PDAC at the primary site and liver, and representative PBRM1 and vimentin staining at the primary site from *Ptf1a*^{CreER}; *LSL-Kras*^{G12D}; *Trp53*$^{f/wt}$ (*ER-KPC*), *Ptf1a*^{CreER}; *LSL-Kras*^{G12D}; *Trp53*$^{f/wt}$; *Pbrm1*$^{f/wt}$ (*ER-KPCPb*$^{+/-}$), and *Ptf1a*^{CreER}; *LSL-Kras*^{G12D}; *Trp53*$^{f/wt}$; *Pbrm1*$^{f/f}$ (*ER-KPCPb*$^{-/-}$) mice. Scale bar, 50 μm. Data are representative of 3 independent experiments.

C ChIP data of the PBRM1 binding region in the vimentin gene promoter and coding regions in the RH-4 rhabdomyosarcoma cell line. TSS: transcription start site.

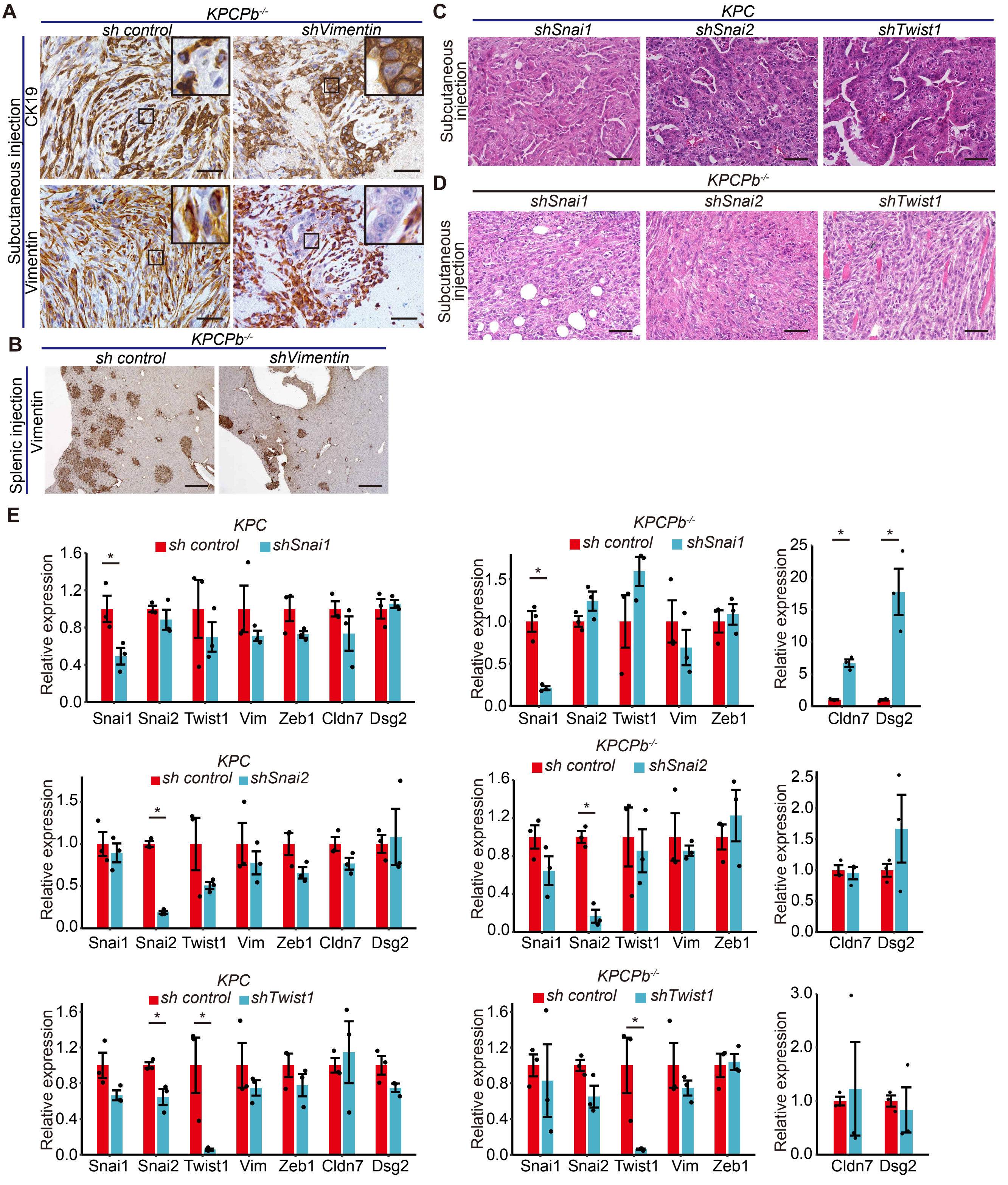

Supplementary Figure 7

A Representative CK19, and vimentin staining in PDAC allografted subcutaneously with *KPCPb-/-shVimentin* and *KPCPb-/-shcontrol*. Scale bar, 50 μm. Data are representative of 3 independent experiments.
B Representative vimentin staining in metastatic PDAC after injection into the spleen with *KPCPb-/-shControl*, and *KPCPb-/-shVimentin* PDAC cells. Scale bar, 500 μm. Data are representative of 3 independent experiments.
C Representative H&E staining in PDACs allografted subcutaneously with *KPCshSnai1*, *KPCshSnai2,* and *KPCshTwist1* PDAC cells. Scale bar, 50 μm. Data are representative of 3 independent experiments.
D Representative H&E staining in PDACs allografted subcutaneously with *KPCPb-/-shSnai1*, *KPCPb-/-shSnai2,* and *KPCPb-/-shTwist1* PDAC cells. Scale bar, 50 μm. Data are representative of 3 independent experiments.
E Quantitative real-time PCR analysis of the relative mRNA expression of *Zeb1*, *Vim*, *Snai1, Snai2, Twist1, Cldn7,* and *Dsg2* in *KPCshSnai1* (n = 3), *KPCshSnai2* (n = 3)*, KPCshTwist1* (n = 3)*, KPCPb-/-shSnai1* (n = 3), *KPCPb-/-shSnai2* (n = 3)*,* and *KPCPb-/-shTwist1* (n = 3) PDAC cells compared to each *shControl*. *$P < 0.05$, Student *t*-test. Data shown as mean ± SE.

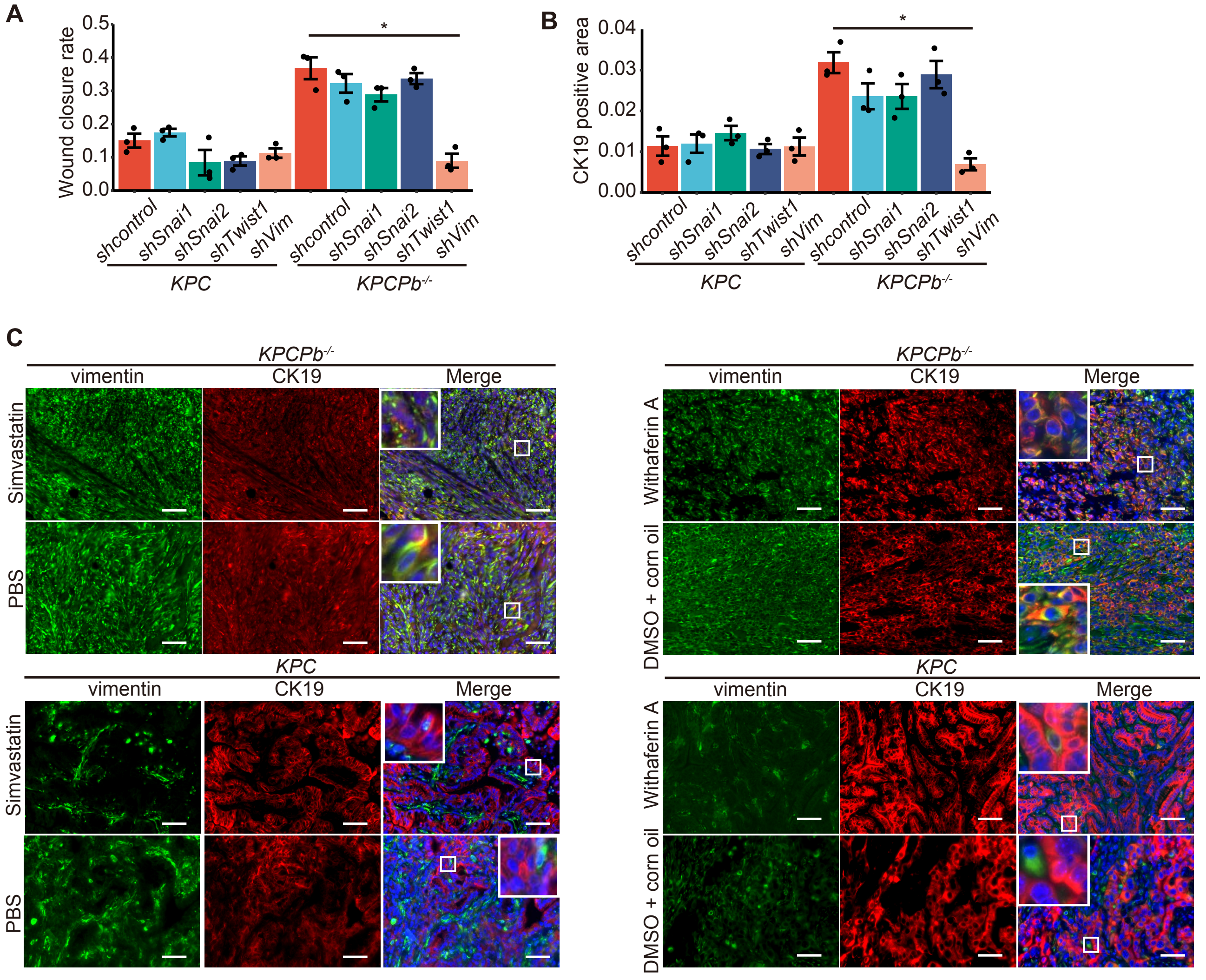

D

	PBRM1 high	PBRM1 low
VIM high	16	42
VIM low	34	13

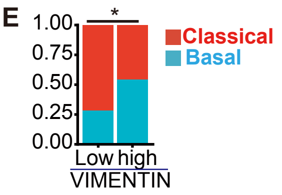

Supplementary Figure 8

A Quantification of the scratch assay with *KPC shControl* (n = 3), *KPCshVimentin* (n = 3), *KPCshSnai1* (n = 3), *KPCshSnai2* (n = 3), *KPCshTwist1* (n = 3), *KPCPb*$^{-/-}$*shControl* (n = 3), *KPCPb*$^{-/-}$*shVimentin* (n = 3), *KPCPb*$^{-/-}$*shSnai1* (n = 3), *KPCPb*$^{-/-}$*shSnai2* (n = 3), and *KPCPb*$^{-/-}$*shTwist1* (n = 3) PDAC cells. *$P < 0.05$, Student *t*-test. Data shown as mean ± SE.

B Quantification of CK19-positive liver metastasis with a splenic injection of *KPC shControl* (n = 3), *KPCshVimentin* (n = 3), *KPCshSnai1* (n = 3), *KPCshSnai2* (n = 3), *KPCshTwist1* (n = 3), *KPCPb*$^{-/-}$*shControl* (n = 3), *KPCPb*$^{-/-}$*shVimentin* (n = 3), *KPCPb*$^{-/-}$*shSnai1* (n = 3), *KPCPb*$^{-/-}$*shSnai2* (n = 3), and *KPCPb*$^{-/-}$*shTwist1* (n = 3) PDAC cells, as determined by combining three independent sections. *$P < 0.05$, Student *t*-test. Data are represented as mean ± SE.

C Representative coimmunostaining of Vimentin, and CK19 in PDAC allografted subcutaneously with *KPCPb*$^{-/-}$ and *KPC* PDAC cells in mice treated with simvastatin, Withaferin A, and each vehicle control. Scale bar, 50 µm. Data are representative of 3 independent experiments.

D Analysis of vimentin expression in surgically resected human PDACs (n = 105) with high (n = 50) or low (n = 55) PBRM1 expression as determined by immunohistochemistry.

E Analysis of molecular subtyping in human PDACs with high (n = 73) and low (n = 74) vimentin expression using the TCGA dataset (n = 147). *$P < 0.05$, Pearson's chi-square test.
